# Adaptive Reinforced Gray Langur Optimization for Feature Selection and SVR Modeling of Polysaccharides in *Dendrobium huoshanense* via NIR Spectroscopy

**DOI:** 10.3390/biomimetics11090604

**Published:** 2026-08-24

**Authors:** Chaochuan Jia, Feilong Yu, Ting Yang, Yu Liu, Maosheng Fu, Fang Wang, Ling Li

**Affiliations:** 1 School of Electronic Information and Artificial Intelligence, West Anhui University, Lu’an 237012, China; 03000076@wxc.edu.cn (C.J.);; 2 Anhui Dabie Mountain Institute of Traditional Chinese Medicine, West Anhui University, Lu’an 237012, China; 3 School of Electrical and Optoelectronic Engineering, West Anhui University, Lu’an 237012, China

**Keywords:** GLO, ARGLO, CEC benchmark, *Dendrobium huoshanense*, polysaccharide content

## Abstract

Adaptive Reinforced Gray Langur Optimization (ARGLO), an enhanced variant of the Gray Langurs Optimizer, is developed for high-dimensional, multimodal, and nonlinear search landscapes susceptible to local trapping. Although the original GLO performs multi-population cooperative search by simulating the social structures of gray langurs, it still suffers from uneven random initialization, insufficient adaptive population partitioning, weak local perturbation, and premature convergence. ARGLO incorporates three strategies: good point set-based oppositional and quasi-oppositional learning initialization, hierarchical equilibrium adaptive population partitioning, and elite-guided hybrid mutation. Collectively, these mechanisms generate a higher-quality starting population, coordinate global search with local refinement, and reduce the risk of entrapment in suboptimal regions. Evidence from component-wise experiments together with the CEC test suite indicates that ARGLO delivers higher solution precision, steadier convergence, as well as more consistent performance, especially as dimensionality increases. Moreover, ARGLO is applied to near-infrared spectral feature selection and SVR parameter optimization for polysaccharide content prediction in *Dendrobium huoshanense*. Compared with unoptimized SVR, ARGLO-SVR reduces RMSE by 35.35% and improves R^2^ by 21.92%; compared with GLO-SVR, it reduces RMSE by 6.05% and improves R^2^ by 2.30%. These results demonstrate the effectiveness and application potential of ARGLO in complex optimization and rapid nondestructive quality detection of traditional Chinese medicinal materials.

## 1. Introduction

Engineering design, machine learning, resource scheduling, path planning, and complex-system modeling frequently involve optimization tasks. Such tasks seek the best feasible decision by optimizing a specified objective while satisfying predefined constraints [1,2,3]. As practical application scenarios become increasingly complex, many optimization problems exhibit high dimensionality, multimodality, nonlinearity, and complex constraints [4,5,6]. These characteristics make traditional mathematical programming methods and gradient-based optimization approaches highly dependent on initial conditions, differentiability assumptions, and local optima, making it difficult to obtain stable and reliable global or near-global optimal solutions within an acceptable computational time [7]. Therefore, metaheuristic optimization algorithms, characterized by stochastic search, population-based cooperation, and independence from gradient information, have become important tools for solving complex optimization problems. Compared with conventional optimization approaches, metaheuristic algorithms generally do not require an explicit mathematical formulation of the problem structure. Owing to their simple implementation, strong adaptability, favorable robustness, and ease of extension, they have been widely employed in continuous combinatorial optimization, engineering design, intelligent modeling and other related tasks [8,9,10,11].

Despite substantial advances, metaheuristic performance remains governed by the coordination of global search and local refinement. Weak global search narrows the explored region and increases the risk of early stagnation at suboptimal points. Conversely, insufficient local refinement delays convergence and limits attainable solution accuracy under a fixed computational budget [12]. Further constraints include loss of population diversity, repetitive search trajectories, vulnerability to parameter choices, and poor scalability with increasing dimensionality. The No Free Lunch theorem implies that algorithmic superiority is task-dependent rather than universal. Consequently, continued research is needed to develop new search mechanisms and tailor existing methods to the structural properties of specific tasks. Existing studies also indicate that many recently developed optimizers focus on enhancing global exploration, improving local exploitation accuracy, alleviating premature convergence, and strengthening adaptability to complex optimization problems [13].

For instance, the Grey Wolf Optimizer (GWO) derives its search mechanism from pack organization and predatory patterns, with alpha, beta, and delta wolves directing candidate updates; it can coordinate exploration and exploitation to some extent, but excessive dependence on leaders may reduce population diversity in high-dimensional complex problems [14]. The Whale Optimization Algorithm (WOA) models the bubble-net feeding strategy of humpback whales, using encircling, spiral movement, and stochastic exploration to update candidate solutions; however, its late-stage exploitation ability and convergence accuracy may be limited by relatively simple search trajectories [15]. Harris Hawks Optimization (HHO) draws on the coordinated hunting tactics of Harris hawks and alternates between global exploration and local refinement using several besiege modes, thereby enabling flexible search behavior; nevertheless, it may still experience stagnation and premature convergence in complex search spaces [16]. The Dung Beetle Optimizer (DBO) derives its search operators from five dung beetle activities: ball rolling, dancing, foraging, stealing, and reproduction, their coordination creates a multi-behavior search framework whose effectiveness partly relies on appropriate transitions between behavioral stages [17]. The Coati Optimization Algorithm (COA) translates the food-seeking and predator-avoidance strategies of coatis into search operators, thereby preserving a moderate level of global exploration, yet it may still face insufficient local exploitation and reduced convergence stability in high-dimensional problems [18]. The Dwarf Mongoose Optimization Algorithm (DMOA) translates the food-searching strategies and role-based social organization of dwarf mongooses into its search operators, where different roles cooperate in the search process; however, its population interaction mechanism may have difficulty maintaining diversity in complex multimodal functions [19]. The Black-winged Kite Algorithm (BKA) mimics the foraging and migratory instincts of black-winged kites to coordinate global exploration with local exploitation; nevertheless, its robustness against local optima stagnation remains insufficient for complex tasks [20]. The Parrot Optimizer (PO) constructs four search operators from parrot foraging, staying, communication, and fear-induced responses. These operators support broad-space sampling and refinement around promising solutions. Despite its behavioral diversity and flexible search pattern, PO remains susceptible to stochastic fluctuations and weak local refinement in complex high-dimensional tasks [21]. The GOOSE algorithm simulates goose migration, flight cooperation, and leader-following mechanisms to conduct optimization search, showing good population cooperation, but population aggregation and inadequate local refinement may still occur in the later search stage [22].

Overall, these algorithms construct effective search frameworks through different natural behaviors, social mechanisms, or population cooperation processes, and they have demonstrated advantages in various optimization tasks. Despite their utility, these algorithms often face significant challenges, such as the trade-off between convergence speed and accuracy, inflexible exploration-exploitation switching, and premature convergence due to reduced diversity. Furthermore, their performance often degrades in high-dimensional landscapes. To address these issues, future research must focus on strategies that enhance initial population quality and dynamic structural adjustment. A primary goal remains boosting local perturbation and high-dimensional stability while preserving the inherent simplicity of the algorithm [23].

Introduced by Saeid Barshandeh et al. in 2026, the Gray Langurs Optimizer (GLO) organizes candidate solutions into multiple interacting populations, its population structure corresponds to three social formations of gray langurs: one-male, multi-male, and all-male groups [24]. In addition, GLO models several behavioral characteristics, including social hierarchy, group migration, dynamic changes in group members, and random roaming within the territory. Compared with single-population optimizers, GLO expands the coverage of the search space through multi-population cooperative search and enhances information exchange by means of inter-group migration. Its effectiveness has been evaluated across classical benchmarks, the CEC2017 test suite, and practical engineering design tasks.

Despite these advantages, the original GLO retains several weaknesses. First, random initialization may produce poorly dispersed candidates as dimensionality increases, thereby reducing the search coverage at the outset. Second, its fixed grouping and migration rules cannot respond effectively to changes in search progress, potentially misallocating computational effort. Third, candidate solutions gradually cluster around the current best regions, eroding population heterogeneity and increasing the likelihood of search stagnation. Finally, the absence of directional local refinement with stronger perturbations restricts recovery from suboptimal basins, particularly on multimodal, hybrid, and high-dimensional landscapes.

To overcome these deficiencies, an enhanced GLO variant, termed Adaptive Reinforced Gray Langur Optimization (ARGLO), is developed with three complementary mechanisms. Although many enhanced metaheuristic algorithms have introduced improved initialization, adaptive parameter control, population regrouping, or mutation operators, these mechanisms are often designed to improve only one stage of the optimization process. Improved initialization can increase the initial diversity of the population but cannot prevent population contraction during later iterations. Adaptive grouping can respond to changes in the search process, but it may not compensate for a poorly distributed initial population. Mutation-based strategies can help restore diversity, yet unguided perturbations may disrupt the exploitation of promising regions. Therefore, an important unresolved challenge is to coordinate initial population construction, dynamic population organization, and stagnation recovery within a single optimization framework.

ARGLO addresses this challenge through the coordinated integration of three complementary mechanisms into the multi-group architecture of GLO. First, good-point-set opposition and quasi-opposition learning constructs a well-dispersed and high-quality starting population by selecting competitive solutions from good-point, opposite, and quasi-opposite candidate sets. Second, hierarchical equilibrium partitioning dynamically reallocates individuals among subpopulations using fitness information and search progress, replacing the fixed population organization of the original GLO with a state-responsive group structure. Third, an elite-guided hybrid mutation operator combines guidance from superior solutions with stronger perturbations, enabling directed local refinement while facilitating escape from stagnant or suboptimal regions. The distinguishing feature of ARGLO is therefore not the simple accumulation of independent operators, but their coordination across different stages of the search. These mechanisms jointly improve initial search coverage, adapt population roles during optimization, and strengthen stagnation recovery during the later search stage.

Accordingly, the principal contributions are outlined as follows:

(1) A good point set-based oppositional and quasi-oppositional learning initialization strategy is proposed to improve the uniformity of the initial population and enhance early-stage global exploration.
(2) A hierarchical equilibrium adaptive population partitioning strategy is designed to dynamically assign search tasks to different individuals according to the population state, thereby improving the balance between exploration and exploitation.
(3) An elite-guided hybrid mutation strategy is developed by introducing a perturbation-based search mechanism to strengthen the algorithm’s ability to escape local optima and perform fine-grained local exploitation.
(4) ARGLO is applied to near-infrared spectral feature selection and SVR parameter optimization for *Dendrobium huoshanense*, and an ARGLO-SVR prediction model is constructed to validate its effectiveness and practical value in real-world applications.

The subsequent sections proceed as follows. Section 2 describes the operating mechanism of GLO, the three proposed modifications, and the complete ARGLO architecture. Section 3 details the experimental protocol and evaluates ARGLO through CEC benchmarks, convergence behavior, statistical comparisons, and ablation experiments. It also examines its use in near-infrared wavelength selection and SVR tuning for *Dendrobium huoshanense*. Section 4 synthesizes the key findings, identifies the remaining limitations, and outlines possible avenues for further investigation.

## 2. Adaptive Reinforced Gray Langur Optimization

The Gray Langurs Optimizer (GLO) maps three typical social structures—one-male, multi-male, and all-male groups—into the optimization search process by simulating the group organization and social behaviors of gray langurs in their natural environment. By integrating behavioral mechanisms such as social hierarchy, individual migration, dynamic changes in group membership, and random activities within the habitat, GLO achieves the iterative updating of candidate solutions. For the specific principles and mathematical models of the GLO algorithm, please refer to Reference [24]. Despite its multi-population architecture, GLO may lose search heterogeneity, stagnate around suboptimal regions, and converge before adequately sampling difficult landscapes. Accordingly, three modifications are incorporated into the GLO framework to broaden search coverage, strengthen fine-scale solution refinement, and produce a steadier convergence process.

### 2.1. Good-Point-Set Opposition and Quasi-Opposition Learning Initialization Strategy

Random initialization in the original GLO can yield poorly dispersed or clustered candidates and becomes less reliable as dimensionality increases. To improve the starting population, a new procedure combining good-point-set sampling with opposition and quasi-opposition learning is introduced. The proposed strategy first employs the theory of good point sets to generate candidate individuals with highly uniform distribution across the search space, as illustrated in Equation (1). Subsequently, it incorporates opposition-based learning and quasi-oppositional learning mechanisms to expand the scope of candidate solutions from multiple directions, including the original positions, symmetric counterparts, and central neighborhoods, as depicted in Equations (2)–(4), thereby generating the initial population individuals, opposition-based population individuals, and quasi-oppositional population individuals, respectively, and forming the candidate population set $P=\{x_{i},x_{i}^{opp},x_{i}^{qopp}\},i=1,2,\ldots \ldots ,N$. Finally, the candidate pool is ranked by fitness, and the best-performing solutions are retained as the starting population $X_{0}$. This selection produces broader spatial coverage and a more balanced candidate distribution, reducing early search stagnation. Consequently, the algorithm begins with stronger search coverage and better solution quality, which facilitates the subsequent optimization process.

(1) g(i,j)=mod(2×cos(2πj/p)•i, 1)

where i=1,2,……,N,j=1,2,……,D, p is a prime satisfying condition p≥2D+3, mod(⋅) denotes the modulo operator.

(2) $$x_{i}=lb+g_{i}\cdot (ub-lb),\;i=1,2,\ldots \ldots ,N$$

(3) $$x_{i}^{opp}=lb+ub-x_{i},\;i=1,2,\ldots \ldots ,N$$

(4) $$x_{i}^{qopp}=C+(2r-1)•|C-x_{i}|$$

where, ub and lb denote the search-space boundaries, C=(lb+ub)/2 gives their midpoint, |•| is the absolute-value operator, and r is sampled uniformly from [0, 1], $g_{i}$ is a vector composed of g(i,j),j=1,2,…D.

### 2.2. Hierarchical Equilibrium Adaptive Population Partitioning Strategy

The original GLO algorithm partitions the population into three distinct groups—one-male group, multi-male group, and all-male group—with a fixed and roughly equal size distribution. While this static partitioning scheme is structurally simple, it fails to account for the algorithm’s current search phase, population diversity, or potential stagnation. Consequently, the search direction cannot adapt to changing demands, from broad-space sampling at the beginning to fine-scale refinement near termination.

Accordingly, a hierarchical equilibrium adaptive population partitioning strategy is incorporated into GLO. It reallocates population shares among the three groups using iteration progress $p_{t}$, population diversity Div, and the search stagnation factor $Stag_{r}$. As defined in Equation (5), $p_{t}$ locates the current search stage by relating iteration t to the maximum iteration count T.

(5) $$p_{t}=t/T$$

Subsequently, population diversity Div is computed using the current population positions *X*, followed by normalization to obtain $Div_{r}$ as defined in Equations (6) and (7).

(6) Div=mean(std(X))

(7) $$Div_{r}=Div/(mean(\max(X)-\min(X))+\varepsilon )$$

Here, $\varepsilon =2.2204\times 10^{-16}$ represents the machine epsilon, introduced to prevent division of $Div_{r}$ by zero.

To quantify whether the algorithm has fallen into search stagnation, the search stagnation factor $Stag_{r}$ is introduced, with its calculation provided in Equation (8).

(8) $$Stag_{r}=\min(stagnation/threshold,1)$$

In this context, stagnation denotes the count of consecutive iterations without improvement in the optimal fitness, and threshold represents the stagnation judgment threshold, as defined in Equation (9).

(9) threshold=max(ceil(0.1⋅T),1)

Building upon these metrics, the algorithm dynamically computes the partition ratios for the three populations. The proportion of the one-male group, denoted as $r_{om}$ (Equation (10)), gradually increases with the iteration process, thereby enhancing local exploitation capabilities in the later stages:

(10) $$r_{om}=0.2+0.3p_{t}+0.1(1-Div_{r})$$

As defined in Equation (11), the multi-male group ratio $r_{mm}$ coordinates broad search with fine-scale solution refinement:

(11) $$r_{mm}=0.45-0.1\cdot Stag_{r}$$

The proportion of the all-male group, $r_{am}$ (Equation (12)), increases alongside *Stag_r_* when the algorithm exhibits stagnation, thus augmenting global search capabilities:

(12) $$r_{am}=1-r_{om}-r_{mm}+0.15\cdot Stag_{r}$$

To prevent any subgroup from becoming excessively small, a minimum ratio constraint is applied (Equation (13)), followed by a normalization process for the three ratios (Equation (14)):

(13) $$r_{k}=\max(r_{k},0.15),k=om,mm,am$$

(14) $$r_{k}=r_{k}/(r_{om}+r_{mm}+r_{am}),k=om,mm,am$$

Finally, the sizes of the three populations are determined based on the normalized ratios, where $N_{om}$, $N_{mm}$ and $N_{am}$ represent the respective scales of the groups, as illustrated in Equations (15)–(17).

(15) $$N_{om}=round(N\cdot r_{om})$$

(16) $$N_{mm}=round(N\cdot r_{mm})$$

(17) $$N_{am}=N-N_{om}-N_{mm}$$

This mechanism reallocates individuals among the three subpopulations according to search progress, population diversity, and stagnation level, thereby coordinating broad-space sampling with fine-scale solution refinement.

### 2.3. Elite-Guided Hybrid Mutation Strategy

Because GLO has no dedicated mutation operator, variation arises mainly from male and juvenile updates, migration, and autonomous roaming. Although these processes preserve basic search activity, differences among candidates shrink as solutions concentrate near the incumbent best, potentially causing early stagnation. An elite-guided hybrid mutation strategy is therefore incorporated by combining elite guidance, differential perturbation, and Student t heavy-tailed perturbation. Elite guidance promotes refinement around promising areas, whereas differential perturbation derives alternative directions from inter-individual differences and helps preserve population heterogeneity. The Student t component permits occasional long-range moves, allowing the search to leave inferior basins when ordinary updates cease to produce improvements.

First, the algorithm selects individuals with higher fitness rankings from the current population to form an elite set. The number of elite individuals is denoted as m=max(2,ceil(0.2⋅N)), and the corresponding elite set is represented as $P_{E}=\{x_{1},x_{2},\ldots ,x_{m}\}$.

Meanwhile, the adaptive mutation probability $p_{m}$ in Equation (18) is linked to the stagnation measure $Stag_{r}$, allowing perturbation frequency to respond to search progress. Larger $p_{m}$ values are assigned at the beginning of the run and whenever solution improvement stalls.

(18) $$p_{m}=0.1+0.25(1-t/T)+0.2\cdot Stag_{r}$$

Next, the algorithm determines whether an individual is a weak individual based on its fitness level within its group. Let the fitness set of the g-th group be $F_{g}$. If an individual satisfies condition $f(x_{i})\geq median(F_{g})$, it is considered a weak individual and is forced to undergo mutation. If condition $f(x_{i})<median(F_{g})$ is satisfied, the individual is regarded as a high-quality solution; in this case, mutation is performed only when a random number $rand1\leq p_{m}$, otherwise the individual is retained unchanged.

Once an individual is selected for mutation, the specific mutation strategy is further determined. If a random number rand2<0.6, an elite-guided differential mutation strategy is adopted, as formulated in Equation (19).

(19) $$v_{i}=x_{i}+F(x_{e}-x_{i})+F(x_{r1}-x_{r2})$$

where $x_{e}\in P_{E}$ denotes an elite individual randomly selected from the elite set, $x_{r1}$ and $x_{r2}$ are two individuals randomly selected from the current population, and F=0.3+0.4(1−t/T) is an adaptive differential scaling factor. On the one hand, the term $F(x_{e}-x_{i})$ guides individuals toward the elite region, enhancing local exploitation capability; on the other hand, $F(x_{r1}-x_{r2})$ introduces differential perturbations to maintain a certain level of global exploration capability.

If an individual is selected for mutation and satisfies rand2≥0.6, a Student t heavy-tailed perturbation mutation is applied, as shown in Equation (20).

(20) $$u_{i}=x_{best}+scale\cdot t_{v}$$

where $x_{best}$ represents the current global best individual, $t_{v}=Z/sqrt(Y/\upsilon )$, Z∼N(0,1), $Y=\sum_{k=1}^{ceil(\upsilon )}z_{k}^{2}$, $z_{k}∼N(0,1)$, $\upsilon =2+8\frac{t}{T}$ is the degree of freedom of the Student’s t distribution, and scale is the perturbation scale defined in Equation (21).

(21) $$scale=[0.02(1-t/T)+0.002+0.02Stag_{r}]\cdot (ub-lb)$$

This strategy leverages the heavy-tailed property of the Student−t distribution to generate larger perturbations during the early search stage or when the algorithm stagnates, thereby improving the ability to escape local optima. As iterations proceed, the degree of freedom gradually increases, and the perturbations become more stable, facilitating fine-grained local search in later stages. Overall, the complete formulation of the strategy is given in Equation (22).

(22) $$x_{i}=\left\{\begin{array}{l} x_{i}\;if(f(x_{i})<median(F_{g})\&\&rand1>p_{m})\\ v_{i}\;if((f(x_{i})\geq median(F_{g})||rand1\leq p_{m})\&\&rand2<0.6)\\ u_{i}\;if((f(x_{i})\geq median(F_{g})||rand1\leq p_{m})\&\&rand2\geq 0.6) \end{array}\right.$$

where median(•) denotes the median operator, both rand1 and rand2 are sampled uniformly from [0, 1].

After mutation, each generated candidate solution is first subjected to boundary constraint handling to ensure that all decision variables remain within the prescribed search range. The fitness of the repaired candidate is then evaluated and compared with that of the corresponding pre-mutation individual. A greedy selection rule is applied: if the candidate solution yields a better fitness value, it is accepted and replaces the original individual; otherwise, the original individual is retained.

**Figure 1:**
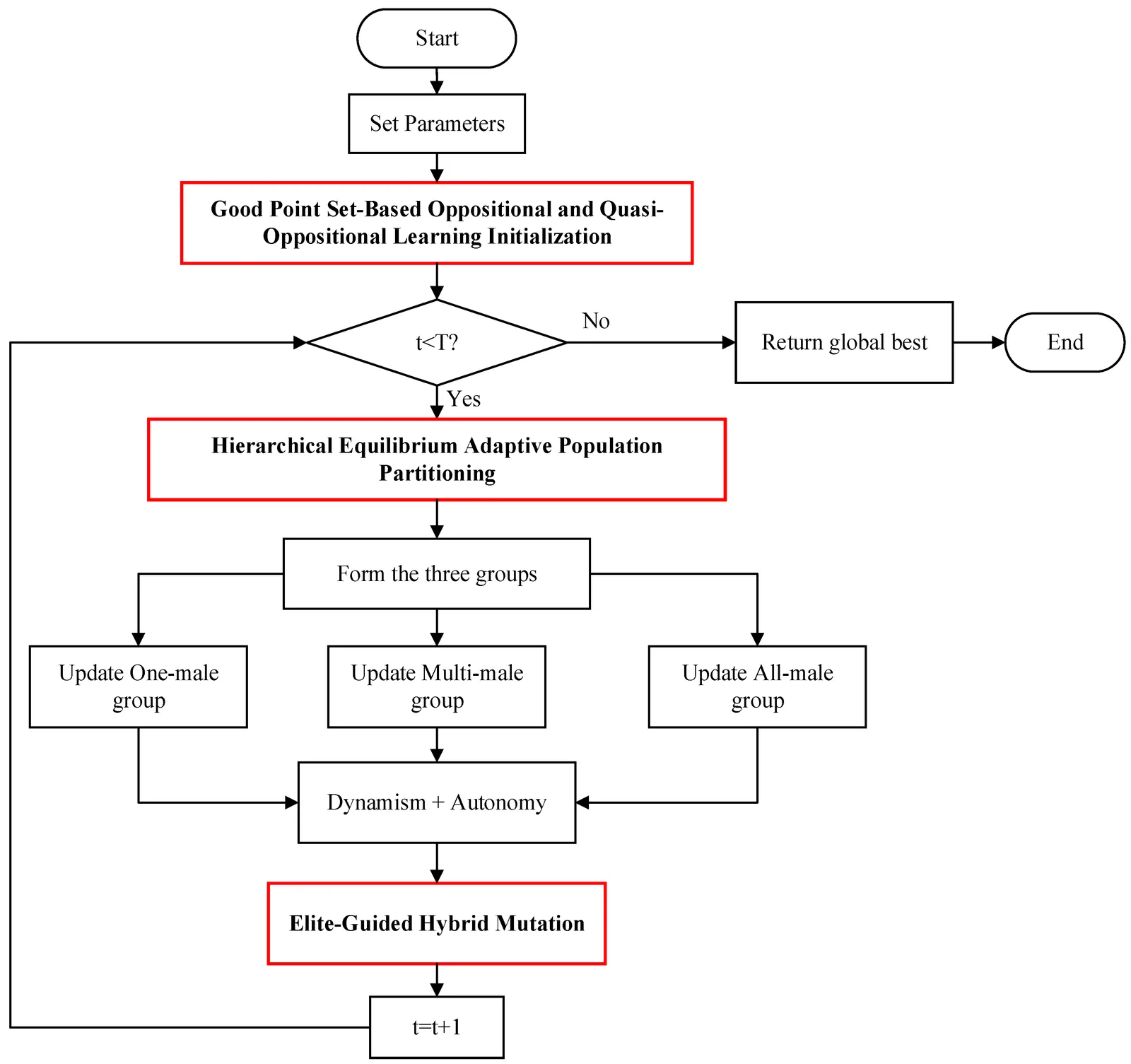
Flowchart of ARGLO.

Through the above mechanisms, the flowchart of ARGLO is shown in Figure 1, ARGLO can preserve high-quality solution structures while enhancing population diversity, and proactively improve its ability to escape local optima when the search process stagnates.

## 3. Experimental Results and Analysis

The evaluation comprises three components: component analysis, benchmark testing, and application assessment. Ablation studies isolate the effects of the three modifications on starting-population quality, search coordination, and final solution precision. ARGLO is then compared with representative metaheuristics across several CEC suites and dimensional settings, with emphasis on accuracy, consistency, and robustness. Finally, ARGLO supports near-infrared wavelength selection and SVR tuning for *Dendrobium huoshanense* polysaccharide prediction, providing an application-level assessment of its practical utility.

### 3.1. Ablation Experiment Design and Effectiveness Verification of Improvement Strategies

**Figure 2:**
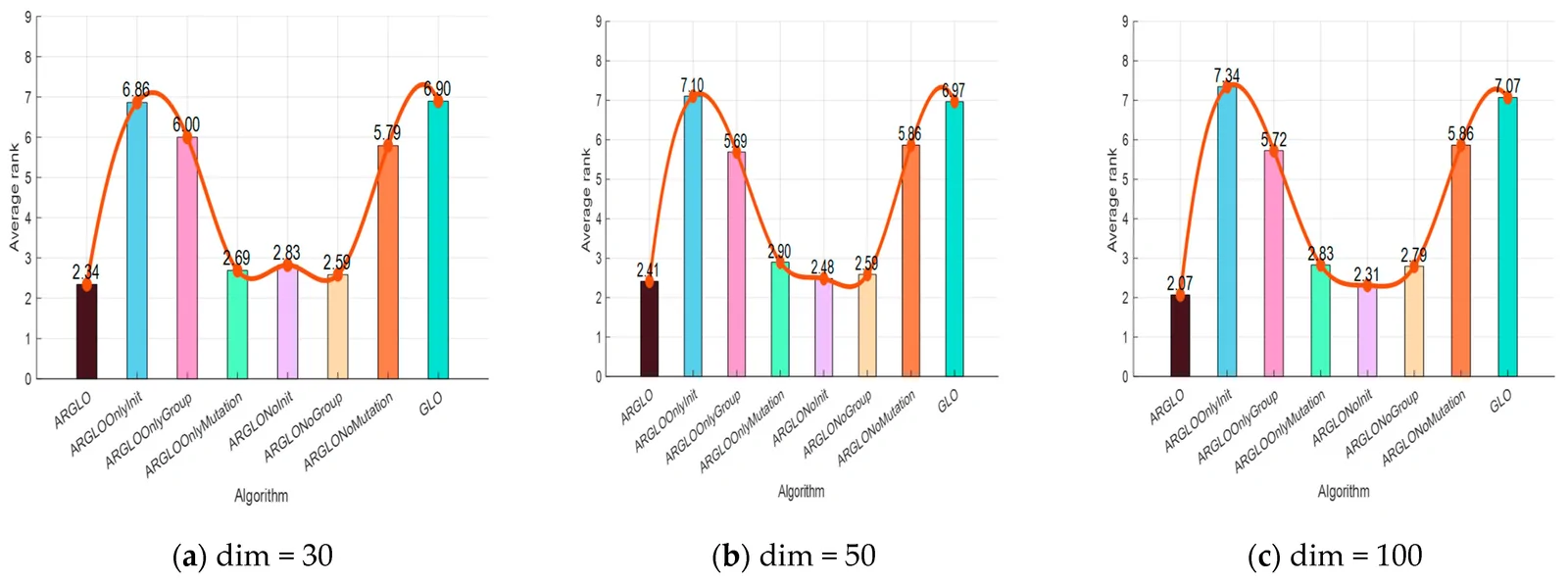
Average ranking diagrams of GLO and 7 variant algorithms on cec2017.

To further examine the contribution and utility of each enhancement, a systematic ablation study is performed by enabling or disabling the good-point-set-based opposition and quasi-opposition learning initialization, the hierarchical equilibrium adaptive population partitioning, and the elite-guided hybrid mutation strategies. The purpose of these experiments is to isolate the impact of each component and to reveal their individual roles as well as their interactions within the overall framework. By comparing the performance of variants with only a single strategy and those with one strategy removed, the study provides a comprehensive understanding of whether each component acts as a core driver, a supportive enhancer, or a collaborative mechanism. Such an analysis not only verifies the rationality of the algorithm design but also clarifies the internal working mechanism of ARGLO, demonstrating how the three strategies work together to improve performance. The ablation study uses the CEC2017 benchmark suite at 30, 50, and 100 dimensions. The three improvement strategies are combined under several configurations to generate different GLO variants, as listed in Table 1. Figure 2 presents the average rankings of these variants.

From the average ranking results across three dimensions (dim = 30, 50, and 100), it can be observed that the contributions of different strategies in ARGLO exhibit clear differences. The full ARGLO model achieves rankings of 2.34, 2.41, and 2.07, respectively, significantly outperforming the original GLO, which obtains 6.90, 6.97, and 7.07, demonstrating that the integration of the three improvement strategies effectively improves the algorithm’s performance.

The analysis of individual strategies shows that using only the good point set-based opposition and quasi-opposition learning initialization strategy (ARGLOOnlyInit) results in the worst performance (6.86, 7.10, 7.34), which is nearly equivalent to the original algorithm, indicating its limited effectiveness when applied alone. Using only the hierarchically balanced adaptive population grouping strategy (ARGLOOnlyGroup) leads to slight improvements (6.00, 5.69, 5.72), but still fails to achieve satisfactory results. In contrast, employing only the elite-guided hybrid mutation strategy (ARGLOOnlyMutation) significantly improves performance (2.69, 2.90, 2.83), approaching that of the full model, which indicates that the mutation strategy has the strongest independent optimization capability.

Further analysis based on removing each strategy reveals that excluding the initialization strategy (ARGLONoInit) causes only a slight performance degradation (2.83, 2.48, 2.31), while removing the grouping strategy (ARGLONoGroup) results in a moderate decline (2.59, 2.59, 2.79). However, removing the mutation strategy (ARGLONoMutation) leads to a substantial deterioration (5.79, 5.86, 5.86), almost reverting to a poor performance level. Overall, the mutation strategy serves as the core driving force for performance improvement in ARGLO, the adaptive grouping strategy provides stable gains, and the initialization strategy, although less effective on its own, further enhances overall performance through interaction with other strategies. Moreover, the advantage of the full model becomes more pronounced as the dimensionality increases.

### 3.2. Multi-Algorithm Comparative Experiments on Multiple Benchmark Function Suites

**Figure 3:**
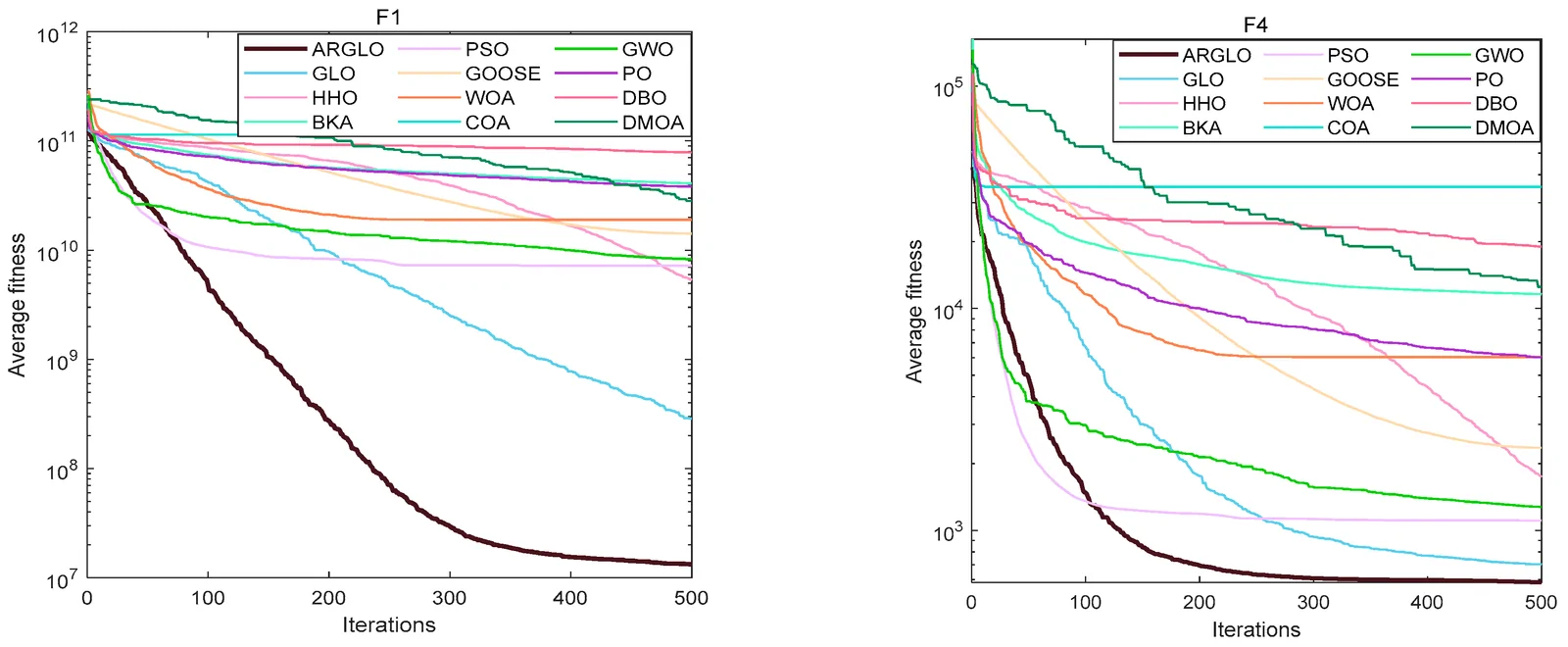

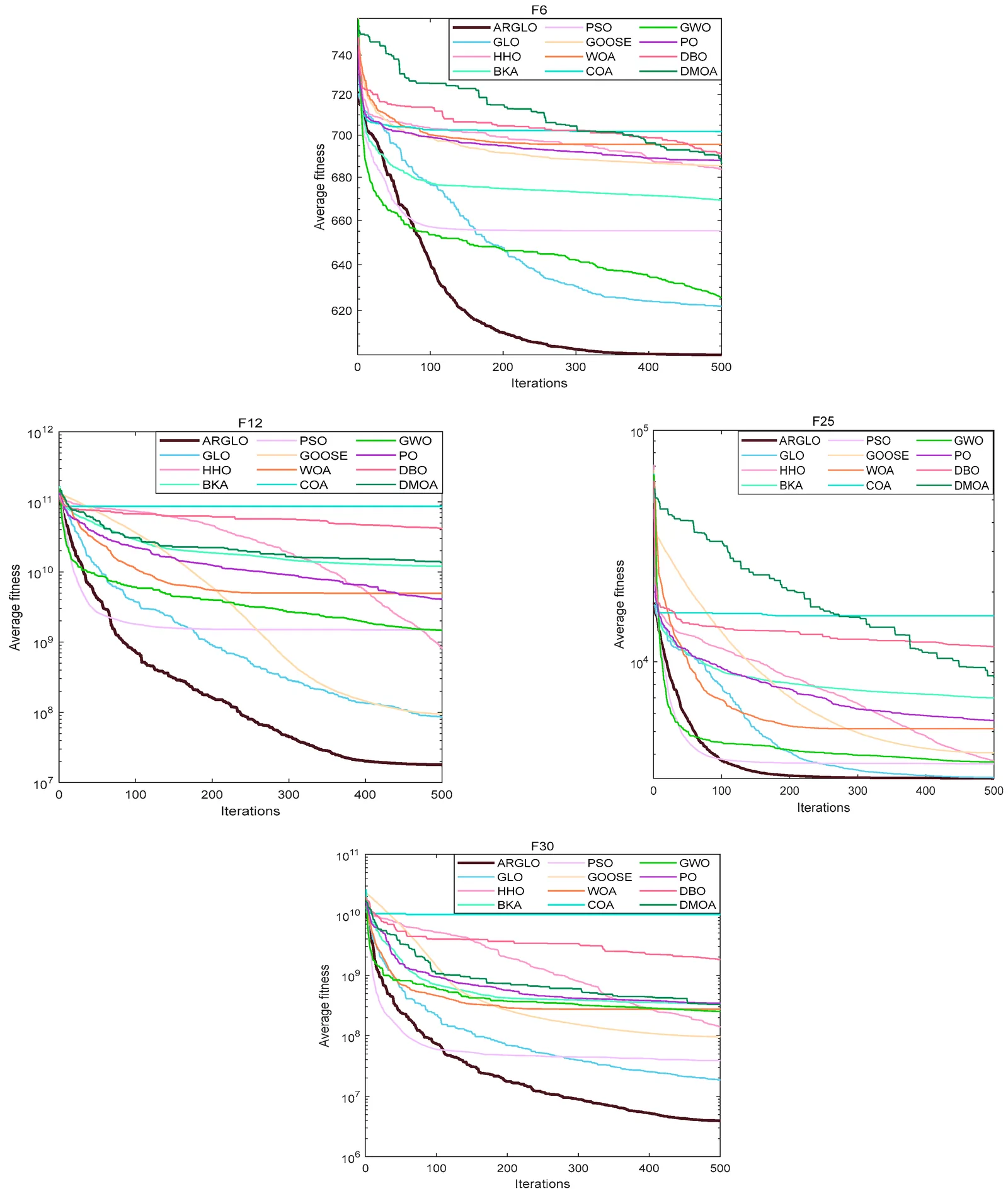
Convergence curves of selected functions.

**Figure 4:**
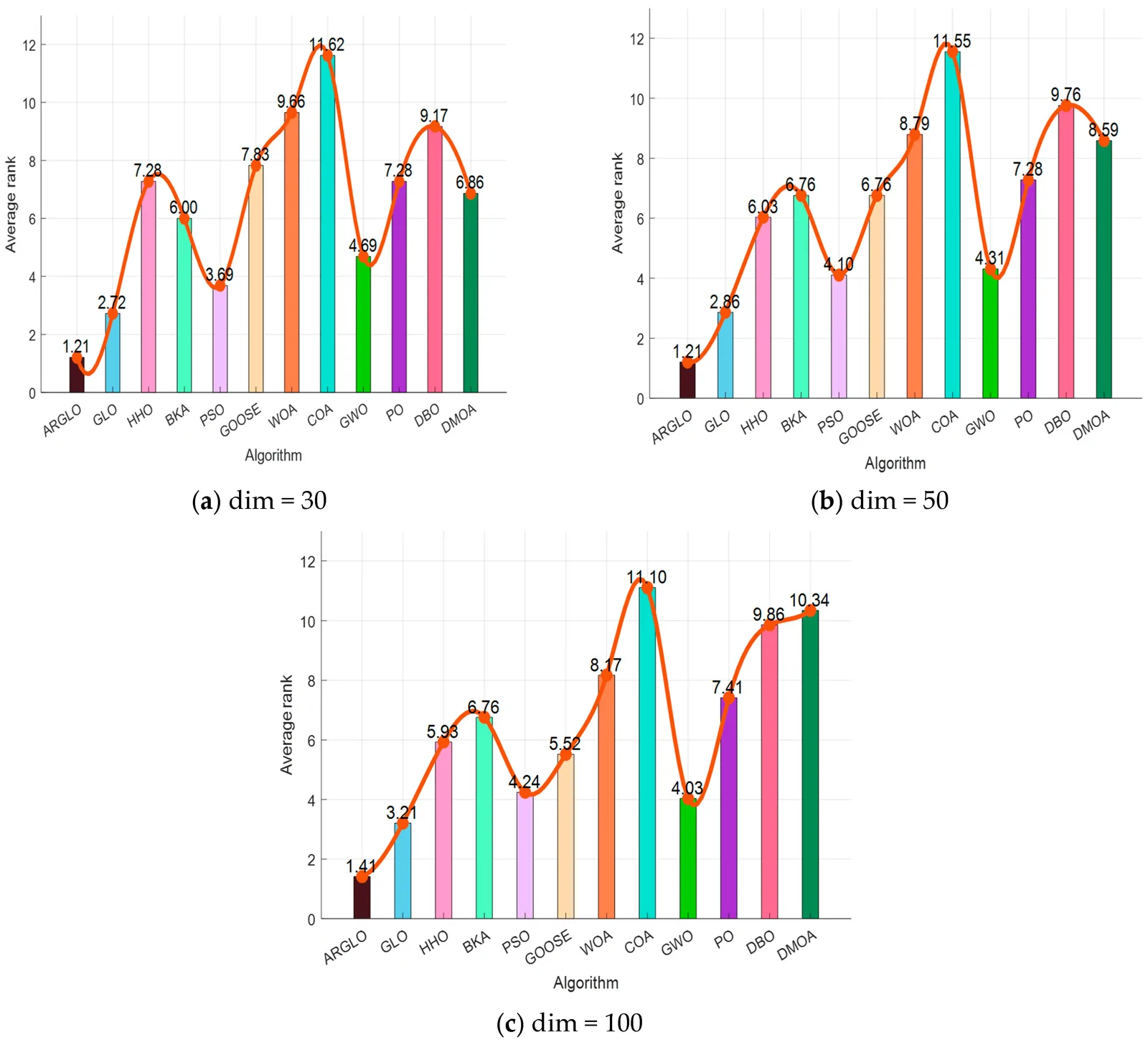
Average ranking diagrams of 12 algorithms on cec2017.

To further assess ARGLO relative to other intelligent optimization algorithms, 11 representative methods, including GLO, HHO, BKA, PSO, GOOSE, WOA, COA, GWO, PO, DBO, and DMOA, were selected as baselines. ARGLO and these algorithms were tested on five benchmark suites, namely CEC2017, CEC2019, CEC2020, CEC2021, and CEC2022, at 30, 50, and 100 dimensions. The comparison examined solution accuracy, convergence behavior, and run-to-run consistency. Under identical experimental settings, every algorithm was executed independently 30 times, and its best, mean, and standard deviation values were recorded. Specifically, the best value reflects the strongest search outcome, the mean value summarizes overall optimization accuracy, and the standard deviation quantifies variability across independent runs. Given the space constraints, Table 2 reports only the results obtained by all algorithms on CEC2017 at 50 dimensions. Figure 3 plots the convergence trajectories for several representative functions, while Figure 4 summarizes the average rankings at 30, 50, and 100 dimensions.

As reported in Table 2, ARGLO performed better overall than the original GLO. Taking the mean value as the evaluation indicator for solution accuracy, ARGLO outperforms GLO on 28 out of 29 valid test functions and is only slightly inferior to GLO on F16. This finding suggests that the three modifications improve the overall solution accuracy of the original GLO. Across the different function categories, ARGLO yields better results for the unimodal and simple multimodal functions, most hybrid functions, and every composition function. This performance pattern indicates improvements in both local refinement and broad search across complex multimodal and high-dimensional coupled landscapes. Furthermore, in terms of standard deviation, ARGLO obtains lower standard deviation values than GLO on 24 functions, indicating that its repeated runs are more stable on most test functions and are less affected by random initialization and search perturbations.

Comparison with the remaining 10 algorithms also favored ARGLO in terms of aggregate optimization performance. According to the mean value indicator, ARGLO ranks first on 25 of the 29 valid functions, corresponding to a winning rate of 86.21%. Specifically, for the unimodal functions, ARGLO obtains the best result on F1. ARGLO ranks first on every simple multimodal function from F4 to F10, indicating that it can effectively address the search interference caused by numerous local optima. For the hybrid functions F11–F20, ARGLO fails to obtain the best result only on F16, while achieving the best results on all the remaining functions. Within the composition group F21–F30, ARGLO does not rank first on F27 and F28 but leads the comparison on all other functions. These findings demonstrate its adaptability and solution accuracy on multimodal, non-separable, strongly coupled problems with increasing dimensionality.

From the perspective of stability, ARGLO obtains the minimum standard deviation on 16 functions, indicating that its results fluctuate less over 30 independent runs and that it has good repeatability. In particular, for hybrid and composition functions, ARGLO not only achieves the best mean values on most functions but also maintains relatively small standard deviations on several complex functions. This suggests that its performance advantage does not arise from occasional successful searches but from stable and reliable optimization behavior. In contrast, although HHO, BKA, PSO, GOOSE, WOA, COA, GWO, PO, DBO, and DMOA achieve competitive results on some individual functions, the number of functions on which they obtain the best results is relatively limited. Moreover, their standard deviations fluctuate more noticeably on complex functions, indicating that these algorithms are more susceptible to local optima and search instability when solving strongly multimodal, strongly coupled, and high-dimensional problems.

The convergence profiles of representative functions in Figure 3 provide further evidence of ARGLO’s faster convergence and lower terminal fitness values. For the unimodal function F1, ARGLO lowers the fitness value sharply during the initial iterations and continues to decline steadily thereafter, indicating effective fine-scale refinement. For the simple multimodal functions F4 and F6, ARGLO descends more rapidly at the outset and keeps improving through the intermediate and final iterations, whereas some comparison algorithms stagnate earlier. This indicates that ARGLO is less susceptible to early search stagnation. For the hybrid function F12, ARGLO follows a steeper overall convergence trajectory and terminates at a lower fitness level, suggesting effective coordination of broad search and local refinement. For the composition functions F25 and F30, ARGLO shows a consistent downward trajectory across the iterations and reaches lower terminal fitness values, indicating reliable performance on high-dimensional composition problems.

According to the average-rank summary in Figure 4, ARGLO ranks first among all algorithms at dim = 30, 50, and 100, with average ranks of 1.21, 1.21, and 1.41, respectively. This ranking pattern indicates consistent performance across different dimensions. In comparison, the average ranks of the original GLO are 2.72, 2.86, and 3.21, respectively, showing a decreasing trend in performance as dimensionality increases. The widening performance gap suggests that the original GLO scales less effectively to high-dimensional and structurally complex search tasks. The gains achieved by ARGLO indicate that the initialization, population partitioning, and mutation strategies improve search efficiency, final solution precision, and run-to-run consistency. In addition, the results on CEC2019, CEC2020, CEC2021, and CEC2022 follow patterns similar to those observed on CEC2017, supporting the consistency and transferability of ARGLO across different benchmark suites.

Let N, D, T, and $C_{f}$ denote the population size, problem dimension, maximum number of iterations, and computational cost of one objective-function evaluation, respectively. The proposed enhancement strategies preserve the asymptotic time complexity of GLO, $O(T(ND+NlogN+NC_{f}))$, but introduce additional constant computational costs. The good-point-set quasi-opposition initialization requires 2N additional objective-function evaluations, whereas the elite-guided hybrid mutation evaluates additional candidate solutions during the iterative search. The stratified adaptive group-division strategy mainly adds diversity calculation, sorting, and regrouping operations. Across the 29 CEC2017 functions at D = 50, the mean runtime over 30 independent runs was 0.9112 s for ARGLO and 0.5661 s for GLO, corresponding to an average runtime overhead of 60.95%.

Despite this additional computational cost, ARGLO achieved superior optimization performance. These results indicate that the additional computational overhead of ARGLO is accompanied by a statistically supported improvement in optimization performance and robustness.

### 3.3. Statistical Analysis Based on the Wilcoxon Rank-Sum Test

To further evaluate the statistical significance of the performance differences, each algorithm was independently executed 30 times on every CEC2017 benchmark function at Dim = 50. The Wilcoxon rank-sum test was then applied separately to the 30 independent results obtained by ARGLO and those obtained by each competing algorithm, with the significance level set to α=0.05. As summarized in Table 3, ARGLO achieved 289 significant wins among the 319 pairwise comparisons, corresponding to an overall significant win rate of 90.6%. In comparison, 27 cases showed no statistically significant difference, and only three cases significantly favored the competing algorithms. Specifically, ARGLO significantly outperformed WOA, COA, and DBO on all 29 functions. It also achieved 28 significant wins against HHO and PO, 27 against DMOA, 26 against GOOSE, 25 against BKA and GWO, 22 against GLO, and 21 against PSO. No significant deterioration was observed against nine of the eleven competing algorithms; the only significant losses occurred against BKA on F3 and DMOA on F27 and F28. Therefore, the Wilcoxon rank-sum test confirms that the improvements achieved by ARGLO are statistically significant and consistent across most of the CEC2017 benchmark functions under the 50-dimensional setting.

### 3.4. Application Verification of ARGLO in Polysaccharide Content Prediction of Dendrobium huoshanenses

To evaluate ARGLO’s practical applicability, ARGLO and GLO were used for near-infrared spectral feature selection and support vector regression (SVR) parameter optimization in *Dendrobium huoshanense* polysaccharide prediction. The models were compared in predictive accuracy, error metrics, and generalization performance.

**Figure 5:**
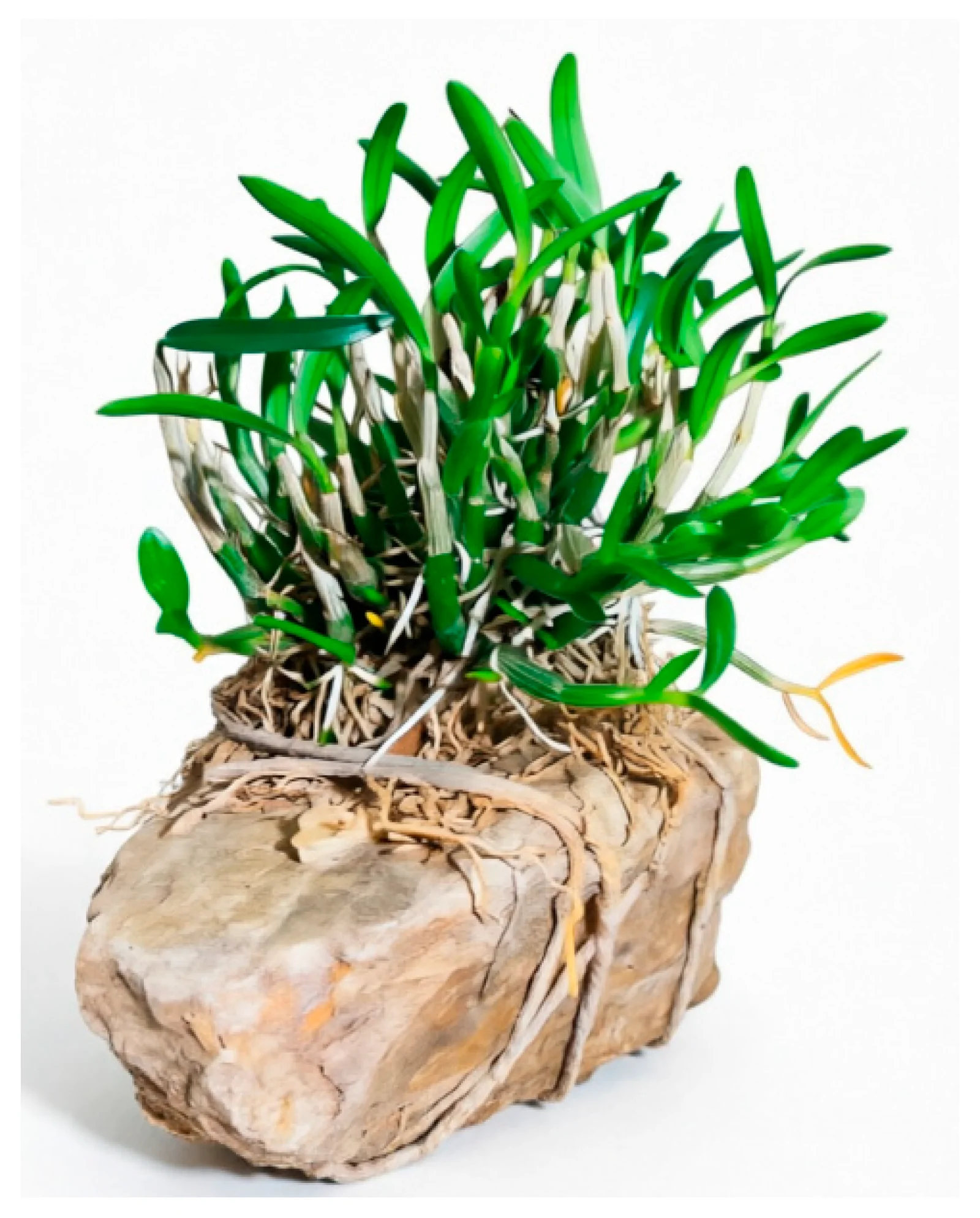
*Dendrobium huoshanense* growing on a rock.

*Dendrobium huoshanense*, shown growing on rocks in Figure 5, is a perennial Orchidaceae herb and a valuable medicinal and edible resource in China. Its polysaccharides are important bioactive constituents and quality indicators, with reported immunomodulatory, antioxidant, anti-inflammatory, hypoglycemic, hepatoprotective, and metabolism-regulating activities [25]. Accurate quantification is therefore important for germplasm screening, processing optimization, quality control, and product standardization.

Polysaccharide quantification generally requires extraction and pretreatment, including hot-water extraction, alcohol precipitation, and ultrasound-, microwave-, or enzyme-assisted extraction. Hot-water extraction followed by ethanol precipitation is widely used because of its simplicity, whereas assisted methods can shorten extraction time and increase yield. Extracted polysaccharides are quantified by phenol–sulfuric acid, anthrone–sulfuric acid, or high-performance liquid chromatography. However, these destructive procedures consume considerable samples and reagents and involve complex pretreatment and long detection cycles. They are therefore unsuitable for rapid batch analysis during the production, circulation, and quality grading of *D. huoshanense* [26].

**Figure 6:**
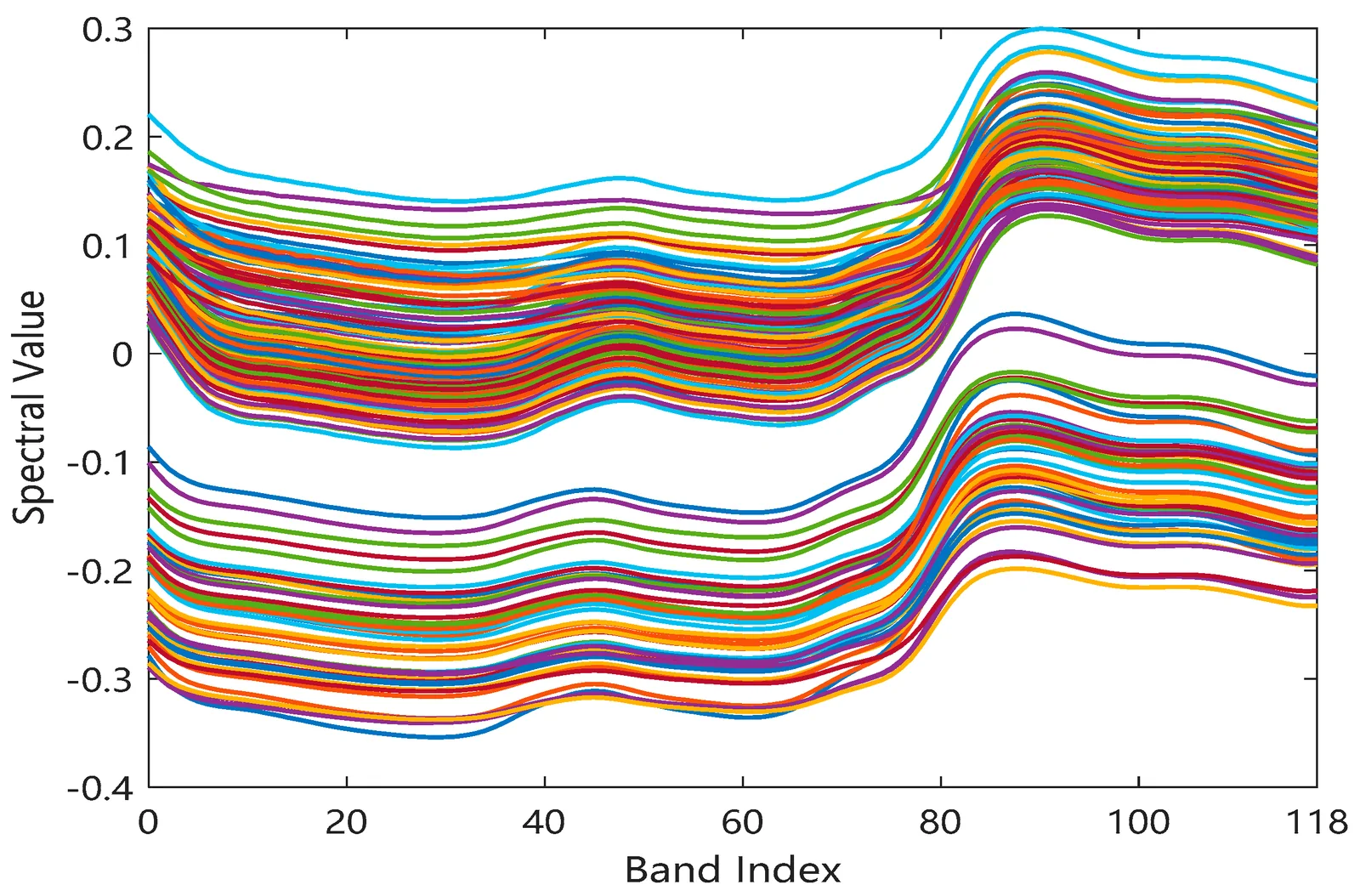
Near-Infrared Spectral Curves of *Dendrobium huoshanense*.

Therefore, a rapid, accurate, and nondestructive method is needed to determine the polysaccharide content of *D. huoshanense*. Unlike conventional physicochemical methods, near-infrared spectroscopy can obtain internal quality information without damaging the sample [27]. Figure 6 presents the near-infrared spectra of *D. huoshanense*. This technique offers rapid measurement, simple operation, environmental compatibility, and potential for online detection, supporting quality monitoring, origin identification, grade assessment, and standardized control. In quantitative near-infrared analysis, rapid nondestructive detection depends on a stable and accurate regression model for polysaccharide prediction. Available modeling approaches comprise partial least squares regression (PLSR), principal component regression (PCR), multiple linear regression (MLR), random forest regression (RFR), support vector regression (SVR), and artificial neural networks. PLSR is commonly adopted in quantitative spectral modeling because it can accommodate multicollinear predictors. However, near-infrared spectra usually contain numerous bands, redundant variables, and substantial noise. Building a PLSR model directly from the full spectrum may increase model complexity, amplify irrelevant information, and weaken its generalization performance.

Spectral feature selection is therefore required to retain informative wavelengths associated with the polysaccharide content of *D. huoshanense*. Removing irrelevant and redundant variables can reduce input dimensionality and improve predictive performance. Support vector regression (SVR) is better suited than linear models to capture nonlinear relationships in small-sample datasets. It can therefore model the complex association between near-infrared spectra and the polysaccharide content of *D. huoshanense*. However, SVR performance is sensitive to the penalty factor and kernel parameter. The former controls the trade-off between training error and model complexity, whereas the latter determines the nonlinear mapping characteristics of the kernel function. Unsuitable parameter values may cause underfitting or overfitting and reduce predictive accuracy and stability. Therefore, selecting informative wavelengths while jointly tuning the key SVR parameters is essential for developing a reliable polysaccharide prediction model.

Intelligent optimization algorithms have the advantages of strong global search capability, high parameter optimization efficiency, and no requirement for gradient information. They have therefore become important tools for regression model parameter optimization and feature selection. Based on this, an improved GLO algorithm is applied in this study to SVR parameter optimization and near-infrared spectral feature selection. Through an intelligent optimization strategy, the optimal feature subset and key SVR parameter combination are searched simultaneously, thereby constructing a rapid nondestructive detection model for polysaccharide content in *D. huoshanense*. This method can effectively reduce the dimensionality of spectral data, decrease the interference of irrelevant and redundant wavelengths, and improve the correlation between selected spectral variables and polysaccharide content. Meanwhile, optimizing SVR parameters enhances the model’s fitting ability for nonlinear relationships and improves its generalization performance. Compared with traditional full-spectrum modeling or empirical parameter selection methods, the improved GLO-SVR modeling approach has stronger adaptability, higher prediction accuracy, and better model stability.

To assess ARGLO for spectral feature selection and SVR parameter tuning, comparative experiments were performed. An SVR model with randomly assigned parameters served as the baseline for examining parameter sensitivity. GLO and ARGLO were then used to select informative wavelengths and tune the SVR parameters, producing the GLO-SVR and ARGLO-SVR models for polysaccharide prediction. Each experiment was independently repeated 30 times, and the mean evaluation metrics are reported in Table 4. Test-set predictions and the convergence profiles of the two optimizers are presented in Figure 3 and Figure 4, respectively.

Table 4 reveals substantial differences among the SVR, GLO-SVR, and ARGLO-SVR models. The baseline SVR yielded relatively poor predictions, with mean absolute error (MAE), mean squared error (MSE), and root mean squared error (RMSE) values of 2.326, 9.024, and 3.004, respectively. Its coefficient of determination (R^2^), residual predictive deviation (RPD), and mean absolute percentage error (MAPE) were 0.73, 1.933, and 0.068, respectively. These results show that randomly assigned SVR parameters were insufficient to model the nonlinear association between near-infrared spectra and the polysaccharide content of *D. huoshanense*, leading to large prediction errors and weak generalization.

In contrast, the performance of the GLO-SVR model is significantly improved after spectral feature selection and SVR parameter optimization using the GLO algorithm. The MAE, MSE, and RMSE are reduced to 1.565, 4.275, and 2.067, respectively, while R^2^ increases to 0.87, RPD increases to 2.81, and MAPE decreases to 0.046. These results demonstrate that the GLO algorithm can select effective spectral features and optimize SVR model parameters to a certain extent, thereby reducing the negative effects of redundant wavelengths and inappropriate parameter settings on model prediction performance.

Further comparison between GLO-SVR and ARGLO-SVR shows that ARGLO-SVR achieves the best results across all evaluation metrics. Specifically, the MAE, MSE, and RMSE of ARGLO-SVR are 1.445, 3.769, and 1.942, respectively, all of which are lower than those of GLO-SVR. Meanwhile, R^2^ and RPD increase to 0.89 and 2.99, respectively, and MAPE further decreases to 0.042. Compared with GLO-SVR, ARGLO-SVR further reduces the error metrics and improves the goodness of fit and prediction capability, indicating that the improved ARGLO algorithm can obtain a more reasonable feature subset and SVR parameter combination, thereby enhancing the prediction accuracy of the model for the polysaccharide content of *D. huoshanense*.

**Figure 7:**
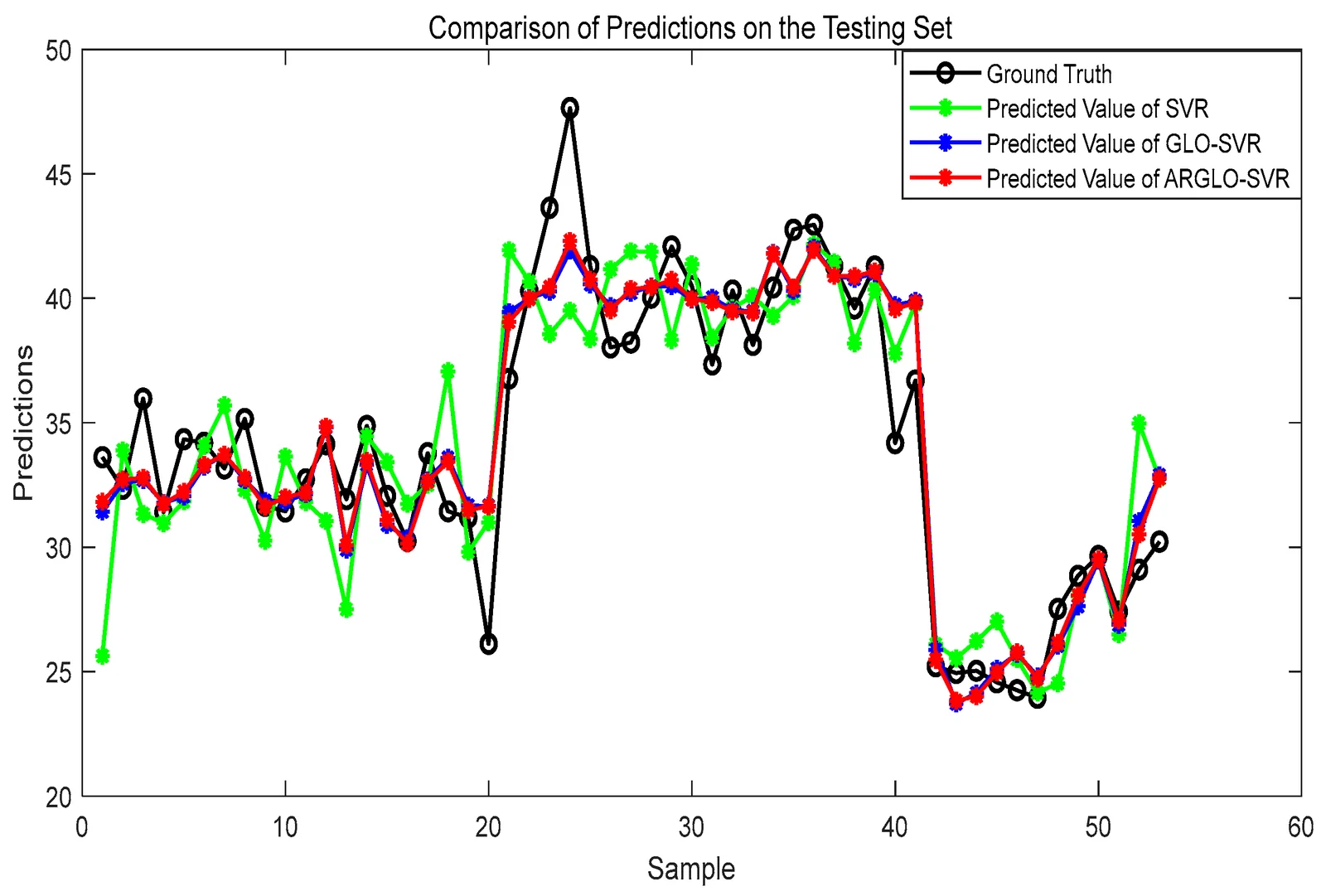
Prediction results of the test set.

As shown in Figure 7, the unoptimized SVR model can roughly capture the overall variation trend of polysaccharide content in *D. huoshanense*, but its prediction curve exhibits relatively large fluctuations and noticeable deviations from the ground truth in several sample regions. After optimization by GLO and ARGLO, the prediction performance of the SVR model is significantly improved. Both GLO-SVR and ARGLO-SVR can better follow the changing trend of the measured values, indicating that spectral feature selection and parameter optimization are effective in improving the nonlinear regression ability of SVR.

Compared with GLO-SVR, the prediction curve of ARGLO-SVR is more consistent with the ground truth curve in most sample intervals. In particular, in regions with relatively sharp changes, such as the rapid increase around samples 20–25, the high-value fluctuation region around samples 25–40, and the sudden decrease around samples 41–43, ARGLO-SVR shows better tracking ability and smaller prediction deviations. In the low-value interval after sample 43, the ARGLO-SVR curve also remains closer to the ground truth and exhibits less fluctuation than the other prediction curves. These results indicate that ARGLO can more effectively optimize the feature subset and SVR parameters, thereby enhancing the fitting accuracy, prediction stability, and generalization capability of the model for polysaccharide content prediction.

**Figure 8:**
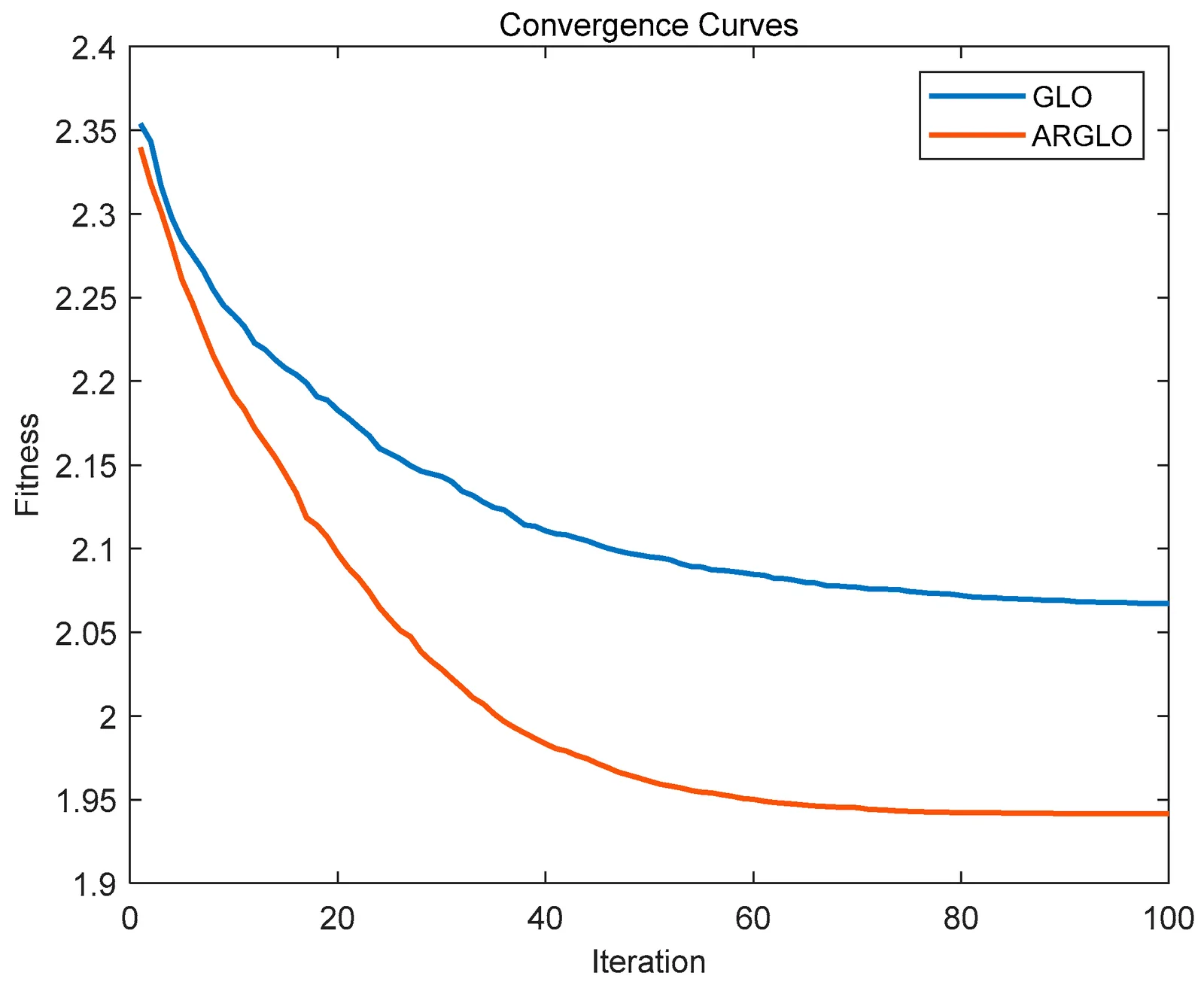
Convergence curves of the two optimization algorithms.

As shown in the convergence curve in Figure 8, the ARGLO algorithm can rapidly reduce the fitness value at the early stage of iteration and maintains a faster decreasing trend throughout the optimization process. In contrast, although the original GLO algorithm can also gradually converge, its convergence speed is relatively slower, and its final fitness value is higher than that of ARGLO. The lower final fitness value of ARGLO indicates that it can more effectively escape local optima during the search process and obtain higher-quality optimization solutions. This demonstrates that ARGLO outperforms the original GLO algorithm in terms of global search capability, local exploitation ability, and convergence accuracy.

In summary, based on the results in Table 4, Figure 7 and Figure 8, the ARGLO-SVR model outperforms both the SVR and GLO-SVR models in terms of error control, fitting accuracy, prediction stability, and convergence performance. Its advantages are mainly reflected in two aspects. On the one hand, ARGLO can more effectively select characteristic wavelengths closely related to the polysaccharide content of *D. huoshanense* from high-dimensional near-infrared spectral data, thereby reducing the interference of irrelevant variables and redundant information. On the other hand, ARGLO can perform more accurate optimization of the key parameters of the SVR model, improving the model’s ability to represent complex nonlinear relationships. Therefore, the ARGLO algorithm has clear advantages in spectral feature selection and SVR parameter optimization, and can effectively improve the prediction accuracy and generalization performance of the near-infrared spectroscopy-based nondestructive detection model for polysaccharide content in *D. huoshanense*.

## 4. Conclusions

This study developed Adaptive Reinforced Gray Langur Optimization (ARGLO) to address limitations in GLO initialization, population partitioning, local perturbation, and convergence behavior. ARGLO integrates three mechanisms into the original framework: good-point-set-based opposition and quasi-opposition learning initialization, hierarchical equilibrium adaptive population partitioning, and elite-guided hybrid mutation. Together, these mechanisms improve starting-population quality and diversity, coordinate broad search with local refinement, and reduce stagnation near suboptimal solutions.

The ablation experiments confirmed that each mechanism contributed to ARGLO performance. The complete ARGLO achieved average ranks of 2.34, 2.41, and 2.07 at dimensions 30, 50, and 100, respectively, outperforming the original GLO. Elite-guided hybrid mutation made the largest independent contribution, whereas adaptive population partitioning provided consistent gains. The initialization mechanism produced limited improvements alone but further enhanced performance when combined with the other two mechanisms, indicating their complementary roles.

Experiments on the CEC2017, CEC2019, CEC2020, CEC2021, and CEC2022 benchmark suites further established the performance of ARGLO against 11 representative optimizers. On the 50-dimensional CEC2017 suite, ARGLO achieved lower mean objective values than GLO on 28 of 29 valid functions. It also produced lower standard deviations on 24 functions, indicating improved solution accuracy and run-to-run consistency. Against the other 10 algorithms, ARGLO ranked first on 25 functions, corresponding to a winning rate of 86.21%, and achieved the lowest standard deviation on 16 functions. Its average ranks at dimensions 30, 50, and 100 were 1.21, 1.21, and 1.41, respectively, placing it first under all three settings. These results indicate that ARGLO performs consistently on multimodal, non-separable, strongly coupled, and high-dimensional problems.

ARGLO was also applied to near-infrared spectral feature selection and SVR parameter tuning for predicting polysaccharide content in *Dendrobium huoshanense*. ARGLO-SVR provided the best predictive results among SVR, GLO-SVR, and ARGLO-SVR. Compared with GLO-SVR, it reduced MAE, MSE, RMSE, and MAPE while increasing R^2^ and RPD to 0.89 and 2.99, respectively. The prediction and convergence profiles also indicated better fitting accuracy, predictive consistency, and convergence behavior. These findings support the use of ARGLO for informative wavelength selection, SVR tuning, and improved model generalization.

Overall, ARGLO improves the solution accuracy, convergence behaviour, consistency, and robustness of the original GLO across the investigated benchmarks and the spectral modelling task. Several limitations nevertheless remain and define the directions of future work. First, integrating three mechanisms raises the per-iteration cost relative to GLO, and this overhead becomes non-negligible at large scale; a systematic complexity and runtime analysis, together with a lighter formulation—for instance, activating the elite-guided mutation only when the stagnation factor exceeds a threshold—would clarify the accuracy-cost trade-off. Second, ARGLO introduces additional control parameters governing the partitioning and mutation schedules, whose sensitivity has not been quantified; self-adaptive or parameter-free settings would improve usability. Third, the present evaluation is confined to continuous, single-objective, unconstrained benchmarks, so extensions to constrained, discrete and binary, multi-objective, dynamic, and large-scale settings remain to be examined—in particular a binary variant tailored to wrapper-based feature selection. Finally, the application study rests on a single medicinal material and a single spectral modality; validation on larger, independently acquired datasets and under cross-batch and cross-instrument conditions is required before practical deployment.

Beyond the present case study, the mechanisms underlying ARGLO are problem-agnostic and transferable to any task that couples high-dimensional multimodal search with model construction. Within near-infrared and hyperspectral analysis, the same wavelength-selection and hyperparameter-tuning pipeline applies to the determination of moisture, alkaloids, flavonoids, and adulterants in other medicinal materials, and extends to agricultural produce grading, food authentication, and pharmaceutical process analytical technology; replacing SVR with PLSR, random forests, extreme learning machines, or lightweight neural networks would allow the modelling stage to be matched to the data at hand. More broadly, ARGLO is applicable to feature selection and hyperparameter optimization in general machine-learning pipelines, to engineering and structural design, path planning and production scheduling, energy-system dispatch and photovoltaic parameter identification, and to multilevel threshold selection in image segmentation. Releasing an open-source implementation would further facilitate independent reproduction and assessment across these domains.

## Figures and Tables

**Table 1:** List including GLO and its variant algorithms.

No	Init	Group	Mutation	Algorithm
1	×	×	×	GLO
2	√	×	×	ARGLOOnlyInit
3	×	√	×	ARGLOOnlyGroup
4	×	×	√	ARGLOOnlyMutation
5	√	√	×	ARGLONoMutation
6	√	×	√	ARGLONoGroup
7	×	√	√	ARGLONoInit
8	√	√	√	ARGLO

Notes: “√” indicates “Yes/Present”, whereas “×” indicates “No/Absent”.

**Table 2:** Results of the 12 algorithms on the cec2017 with dim = 50.

	Algorithms	ARGLO	GLO	HHO	BKA	PSO	GOOSE	WOA	COA	GWO	PO	DBO	DMOA
Functions													
F1	Best	6.5237 × 10^6^	2.5653 × 10^8^	3.7996 × 10^9^	3.0134 × 10^10^	4.2805 × 10^9^	1.1455 × 10^9^	1.4358 × 10^10^	1.0178 × 10^11^	6.4419 × 10^9^	2.3319 × 10^10^	7.1569 × 10^10^	1.9430 × 10^10^
	Std	6.3023 × 10^6^	4.1168 × 10^7^	1.2661 × 10^9^	8.8595 × 10^9^	3.0838 × 10^9^	1.3614 × 10^10^	4.5241 × 10^9^	8.2366 × 10^9^	1.4662 × 10^9^	1.0266 × 10^10^	4.8792 × 10^9^	6.9298 × 10^9^
	Avg	1.3303 × 10^7^	2.8657 × 10^8^	5.3159 × 0^9^	4.0875 × 10^10^	7.1891 × 10^9^	1.4178 × 10^10^	1.8974 × 10^10^	1.1439 × 10^11^	8.2981 × 10^9^	3.8194 × 10^10^	7.8408 × 10^10^	2.8184 × 10^10^
F3	Best	1.2734 × 10^5^	1.5298 × 10^5^	1.4406 × 0^5^	7.3887 × 10^4^	1.2142 × 10^5^	3.7143 × 10^5^	1.7827 × 10^5^	1.6707 × 10^5^	1.5301 × 10^5^	9.9939 × 10^4^	1.6591 × 10^5^	8.4416 × 10^5^
	Std	1.4470 × 10^4^	1.7770 × 10^4^	2.1556 × 0^4^	9.5905 × 10^3^	6.5985 × 10^4^	4.5455 × 10^4^	1.2584 × 10^5^	1.9167 × 10^4^	2.9284 × 10^4^	3.3779 × 10^4^	8.0588 × 10^4^	3.0747 × 10^5^
	Avg	1.4299 × 10^5^	1.8050 × 10^5^	1.6790 × 0^5^	8.4737 × 10^4^	2.1019 × 10^5^	4.2599 × 10^5^	2.7026 × 10^5^	1.9043 × 10^5^	1.8312 × 10^5^	1.3851 × 10^5^	2.4214 × 10^5^	1.0513 × 10^6^
F4	Best	5.0346 × 10^2^	6.6931 × 10^2^	1.4521 × 0^3^	3.4537 × 10^3^	8.6828 × 10^2^	8.7144 × 10^2^	4.5311 × 10^3^	2.5544 × 10^4^	9.3155 × 10^2^	4.4042 × 10^3^	1.6690 × 10^4^	7.9668 × 10^3^
	Std	7.0787 × 10^1^	3.4481 × 10^1^	2.6928 × 0^2^	1.2849 × 10^4^	2.9406 × 10^2^	2.1735 × 10^3^	2.1393 × 10^3^	7.5488 × 10^3^	3.3363 × 10^2^	1.7434 × 10^3^	2.7558 × 10^3^	4.7045 × 10^3^
	Avg	5.8349 × 10^2^	7.0350 × 10^2^	1.7433 × 0^3^	1.1580 × 10^4^	1.1101 × 10^3^	2.3577 × 10^3^	6.0269 × 10^3^	3.5222 × 10^4^	1.2783 × 10^3^	6.0180 × 10^3^	1.8957 × 10^4^	1.2494 × 10^4^
F5	Best	6.1763 × 10^2^	7.4918 × 10^2^	9.0988 × 0^2^	8.3309 × 10^2^	7.9542 × 10^2^	8.7521 × 10^2^	1.0075 × 10^3^	1.1604 × 10^3^	7.2602 × 10^2^	9.7712 × 10^2^	1.0594 × 10^3^	1.1012 × 10^3^
	Std	1.0041 × 10^2^	3.7086 × 10^1^	1.7084 × 0^1^	1.2984 × 10^2^	2.0392 × 10^1^	2.1041 × 10^2^	8.3600 × 10^1^	2.5753 × 10^1^	4.5504 × 10^1^	3.1659 × 10^1^	4.2152 × 10^1^	3.0711 × 10^1^
	Avg	6.8320 × 10^2^	7.8655 × 10^2^	9.3477 × 0^2^	9.6196 × 10^2^	8.2815 × 10^2^	1.0376 × 10^3^	1.0738 × 10^3^	1.1903 × 10^3^	7.7284 × 10^2^	1.0248 × 10^3^	1.1206 × 10^3^	1.1484 × 10^3^
F6	Best	6.0084 × 10^2^	6.1458 × 10^2^	6.7529 × 0^2^	6.6128 × 10^2^	6.4724 × 10^2^	6.6776 × 10^2^	6.8747 × 10^2^	6.9527 × 10^2^	6.1732 × 10^2^	6.8121 × 10^2^	6.8679 × 10^2^	6.7131 × 10^2^
	Std	4.1640 × 10^−1^	4.4929 × 10^0^	5.1025 × 0^0^	5.9591 × 10^0^	5.4361 × 10^0^	1.3421 × 10^1^	7.0793 × 10^0^	4.1995 × 10^0^	5.7867 × 10^0^	5.6192 × 10^0^	4.0517 × 10^0^	9.6122 × 10^0^
	Avg	6.0124 × 10^2^	6.2187 × 10^2^	6.8370 × 0^2^	6.6931 × 10^2^	6.5522 × 10^2^	6.8541 × 10^2^	6.9564 × 10^2^	7.0183 × 10^2^	6.2552 × 10^2^	6.8796 × 10^2^	6.9126 × 10^2^	6.8642 × 10^2^
F7	Best	1.0613 × 10^3^	1.1335 × 10^3^	1.8771 × 0^3^	1.6352 × 10^3^	1.1819 × 10^3^	4.7284 × 10^3^	1.7524 × 10^3^	1.9157 × 10^3^	1.0535 × 10^3^	1.7063 × 10^3^	1.7139 × 10^3^	1.6184 × 10^3^
	Std	1.6308 × 10^1^	5.7406 × 10^1^	3.3838 × 0^1^	7.3131 × 10^1^	1.6812 × 10^2^	3.5728 × 10^2^	1.1681 × 10^2^	9.0650 × 10^1^	9.7348 × 10^1^	7.8207 × 10^1^	3.8940 × 10^1^	1.2547 × 10^2^
	Avg	1.0833 × 10^3^	1.2147 × 10^3^	1.9036 × 0^3^	1.7024 × 10^3^	1.3220 × 10^3^	5.1659 × 10^3^	1.8808 × 10^3^	2.0439 × 10^3^	1.1302 × 10^3^	1.8350 × 10^3^	1.7694 × 10^3^	1.7948 × 10^3^
F8	Best	9.2069 × 10^2^	1.0622 × 10^3^	1.2066 × 10^3^	1.1011 × 10^3^	1.0701 × 10^3^	1.2508 × 10^3^	1.3288 × 10^3^	1.4219 × 10^3^	1.0205 × 10^3^	1.3521 × 10^3^	1.3873 × 10^3^	1.4508 × 10^3^
	Std	2.5347 × 10^1^	3.9199 × 10^1^	3.3993 × 10^1^	6.3103 × 10^1^	4.0868 × 10^1^	1.4026 × 10^2^	5.3912 × 10^1^	5.6054 × 10^1^	4.3343 × 10^1^	2.5822 × 10^1^	1.5395 × 10^1^	2.1344 × 10^1^
	Avg	9.4810 × 10^2^	1.1217 × 10^3^	1.2367 × 10^3^	1.1811 × 10^3^	1.1402 × 10^3^	1.4026 × 10^3^	1.3923 × 10^3^	1.4787 × 10^3^	1.0809 × 10^3^	1.3793 × 10^3^	1.3999 × 10^3^	1.4756 × 10^3^
F9	Best	9.1244 × 10^2^	1.3665 × 10^4^	2.5941 × 10^4^	1.1883 × 10^4^	9.4168 × 10^3^	1.4449 × 10^4^	2.6288 × 10^4^	3.5042 × 10^4^	5.9784 × 10^3^	1.9925 × 10^4^	3.0917 × 10^4^	4.3221 × 10^4^
	Std	1.2738 × 10^2^	6.3806 × 10^3^	3.0838 × 10^3^	3.0174 × 10^3^	1.1778 × 10^3^	5.2056 × 10^3^	1.3647 × 10^4^	3.1246 × 10^3^	7.7690 × 10^3^	3.5084 × 10^3^	4.8727 × 10^3^	8.4754 × 10^3^
	Avg	9.9196 × 10^2^	1.8899 × 10^4^	3.0208 × 10^4^	1.6076 × 10^4^	1.0794 × 10^4^	1.7038 × 10^4^	4.1823 × 10^4^	3.9456 × 10^4^	1.4633 × 10^4^	2.4961 × 10^4^	3.5076 × 10^4^	5.7678 × 10^4^
F10	Best	6.0339 × 10^3^	8.8554 × 10^3^	9.4935 × 10^3^	9.1111 × 10^3^	6.3059 × 10^3^	8.1349 × 10^3^	1.0784 × 10^4^	1.5049 × 10^4^	7.3755 × 10^3^	1.1489 × 10^4^	1.2847 × 10^4^	1.5594 × 10^4^
	Std	5.6337 × 10^2^	7.4966 × 10^2^	8.2381 × 10^2^	2.0160 × 10^3^	9.5203 × 10^2^	9.0127 × 10^2^	1.7620 × 10^3^	3.2229 × 10^2^	3.8242 × 10^3^	6.1023 × 10^2^	1.1260 × 10^3^	3.7310 × 10^2^
	Avg	6.7627 × 10^3^	9.5345 × 10^3^	1.0597 × 10^4^	1.0379 × 10^4^	7.6850 × 10^3^	9.0886 × 10^3^	1.3057 × 10^4^	1.5427 × 10^4^	1.1110 × 10^4^	1.2195 × 10^4^	1.4027 × 10^4^	1.6101 × 10^4^
F11	Best	1.3740 × 10^3^	2.4302 × 10^3^	2.3142 × 10^3^	3.2110 × 10^3^	1.5115 × 10^3^	1.5171 × 10^3^	7.0496 × 10^3^	2.4299 × 10^4^	4.8552 × 10^3^	3.0519 × 10^3^	1.6569 × 10^4^	5.8070 × 10^4^
	Std	3.1310 × 10^1^	4.4877 × 10^2^	2.7463 × 10^2^	1.0630 × 10^3^	1.6432 × 10^2^	2.2704 × 10^3^	2.6513 × 10^3^	2.0722 × 10^3^	1.8216 × 10^3^	2.5051 × 10^3^	9.6652 × 10^2^	1.9959 × 10^4^
	Avg	1.4064 × 10^3^	2.7783 × 10^3^	2.7231 × 10^3^	4.3974 × 10^3^	1.7137 × 10^3^	4.1028 × 10^3^	8.9981 × 10^3^	2.7219 × 10^4^	6.3083 × 10^3^	5.1955 × 10^3^	1.8202 × 10^4^	7.9456 × 10^4^
F12	Best	8.1604 × 10^6^	7.8456 × 10^7^	2.8373 × 10^8^	8.7923 × 10^8^	6.2835 × 10^8^	4.9140 × 10^7^	3.3780 × 10^9^	6.2031 × 10^10^	2.6771 × 10^8^	7.8360 × 10^8^	3.5575 × 10^10^	8.2285 × 10^9^
	Std	8.8536 × 10^6^	1.3885 × 10^7^	4.3079 × 10^8^	2.0391 × 10^10^	1.1293 × 10^9^	2.8375 × 10^7^	1.4849 × 10^9^	2.2194 × 10^10^	1.5908 × 10^9^	2.4982 × 10^9^	5.5028 × 10^9^	4.2477 × 10^9^
	Avg	1.7937 × 10^7^	8.6043 × 10^7^	8.1314 × 10^8^	1.2093 × 10^10^	1.4962 × 10^9^	9.5352 × 10^7^	4.9439 × 10^9^	8.6150 × 10^10^	1.4752 × 10^9^	4.0896 × 10^9^	4.1166 × 10^10^	1.3628 × 10^10^
F13	Best	1.0215 × 10^4^	1.0311 × 10^6^	1.2276 × 10^7^	1.3185 × 10^7^	4.2370 × 10^4^	1.2234 × 10^5^	1.5784 × 10^8^	5.7874 × 10^10^	1.1030 × 10^8^	5.4815 × 10^7^	1.4524 × 10^10^	1.1164 × 10^8^
	Std	9.9202 × 10^3^	1.0297 × 10^6^	9.7297 × 10^6^	1.1976 × 10^10^	1.3599 × 10^9^	6.4975 × 10^4^	2.0787 × 10^8^	1.0281 × 10^10^	1.1376 × 10^8^	1.1487 × 10^8^	4.2035 × 10^9^	4.9480 × 10^7^
	Avg	1.8911 × 10^4^	2.0089 × 10^6^	2.6308 × 10^7^	5.4607 × 10^9^	1.1358 × 10^9^	1.8573 × 10^5^	4.1039 × 10^8^	7.1730 × 10^10^	2.4689 × 10^8^	1.4019 × 10^8^	1.9791 × 10^10^	1.7582 × 10^8^
F14	Best	8.7948 × 10^4^	1.5777 × 10^5^	9.1865 × 10^5^	4.6020 × 10^4^	2.7284 × 10^4^	3.6328 × 10^4^	3.1456 × 10^5^	1.1417 × 10^8^	5.2980 × 10^5^	1.1967 × 10^6^	1.8731 × 10^6^	1.9891 × 10^6^
	Std	1.6208 × 10^5^	9.9898 × 10^5^	2.4239 × 10^6^	7.3872 × 10^6^	4.6640 × 10^5^	3.0293 × 10^5^	4.6720 × 10^6^	7.9554 × 10^7^	7.8953 × 10^6^	2.3559 × 10^6^	4.1979 × 10^6^	4.4597 × 10^6^
	Avg	2.7375 × 10^5^	1.0896 × 10^6^	4.2083 × 10^6^	3.4763 × 10^6^	3.5888 × 10^5^	2.7534 × 10^5^	4.9501 × 10^6^	1.6225 × 10^8^	4.3059 × 10^6^	4.3848 × 10^6^	8.7234 × 10^6^	7.2145 × 10^6^
F15	Best	9.8875 × 10^3^	4.1482 × 10^4^	7.3305 × 10^5^	7.3737 × 10^4^	1.6405 × 10^4^	1.5438 × 10^4^	1.0775 × 10^7^	5.4960 × 10^9^	6.7109 × 10^4^	7.0672 × 10^5^	3.1969 × 10^8^	2.9268 × 10^7^
	Std	5.0939 × 10^3^	1.4828 × 10^5^	3.2880 × 10^5^	3.9694 × 10^9^	1.5433 × 10^4^	6.6588 × 10^3^	2.3081 × 10^7^	2.9951 × 10^9^	3.2413 × 10^7^	1.5158 × 10^8^	1.1102 × 10^9^	1.6495 × 10^7^
	Avg	1.7281 × 10^4^	1.9436 × 10^5^	1.2181 × 10^6^	1.7755 × 10^9^	3.1080 × 10^4^	1.9876 × 10^4^	2.6046 × 10^7^	8.0999 × 10^9^	1.5335 × 10^7^	1.1467 × 10^8^	1.9886 × 10^9^	4.0484 × 10^7^
F16	Best	2.9461 × 10^3^	2.6109 × 10^3^	3.5838 × 10^3^	3.2861 × 10^3^	3.2938 × 10^3^	3.8568 × 10^3^	5.7125 × 10^3^	8.2075 × 10^3^	2.5835 × 10^3^	5.1183 × 10^3^	5.6629 × 10^3^	6.1530 × 10^3^
	Std	3.7409 × 10^2^	3.6717 × 10^2^	7.6551 × 10^2^	3.3852 × 10^2^	3.1565 × 10^2^	8.1271 × 10^2^	4.7142 × 10^2^	2.9738 × 10^3^	4.8666 × 10^2^	8.4717 × 10^2^	4.2693 × 10^2^	5.3841 × 10^2^
	Avg	3.4123 × 10^3^	2.9842 × 10^3^	4.7453 × 10^3^	3.8513 × 10^3^	3.6945 × 10^3^	4.8939 × 10^3^	6.2287 × 10^3^	1.0868 × 10^4^	3.2386 × 10^3^	5.8374 × 10^3^	6.1373 × 10^3^	6.9279 × 10^3^
F17	Best	2.3648 × 10^3^	2.6050 × 10^3^	3.5872 × 10^3^	3.2287 × 10^3^	3.0136 × 10^3^	3.4776 × 10^3^	3.5427 × 10^3^	4.6076 × 10^3^	2.7768 × 10^3^	3.8362 × 10^3^	3.6556 × 10^3^	5.4027 × 10^3^
	Std	1.2440 × 10^2^	3.6554 × 10^2^	3.5633 × 10^2^	1.0934 × 10^3^	2.6436 × 10^2^	3.8002 × 10^2^	1.1064 × 10^3^	8.9620 × 10^3^	1.3302 × 10^2^	6.4575 × 10^2^	9.1480 × 10^2^	2.0659 × 10^2^
	Avg	2.5582 × 10^3^	3.0887 × 10^3^	4.0609 × 10^3^	4.0438 × 10^3^	3.3945 × 10^3^	3.8356 × 10^3^	4.8422 × 10^3^	9.1945 × 10^3^	2.9519 × 10^3^	4.4187 × 10^3^	5.1002 × 10^3^	5.5932 × 10^3^
F18	Best	2.1929 × 10^5^	9.9481 × 10^5^	4.5756 × 10^6^	4.6367 × 10^5^	2.6560 × 10^5^	7.0590 × 10^5^	1.9957 × 10^7^	6.6756 × 10^7^	1.1956 × 10^6^	2.8509 × 10^6^	6.5154 × 10^6^	7.9330 × 10^7^
	Std	6.6452 × 10^5^	1.8324 × 10^6^	7.9452 × 10^6^	9.2940 × 10^5^	5.7659 × 10^6^	7.4447 × 10^5^	7.4084 × 10^7^	1.6552 × 10^8^	5.2376 × 10^6^	2.2360 × 10^7^	1.4121 × 10^7^	4.5409 × 10^7^
	Avg	1.0693 × 10^6^	3.0166 × 10^6^	1.1405 × 10^7^	1.6244 × 10^6^	4.6444 × 10^6^	1.3183 × 10^6^	6.8952 × 10^7^	2.8754 × 10^8^	4.7359 × 10^6^	2.5019 × 10^7^	2.2821 × 10^7^	1.3291 × 10^8^
F19	Best	1.3582 × 10^4^	3.9614 × 10^4^	1.1027 × 10^6^	6.3984 × 10^5^	1.3089 × 10^4^	2.2551 × 10^5^	1.1290 × 10^7^	2.0570 × 10^9^	3.1563 × 10^5^	3.2990 × 10^6^	5.5375 × 10^8^	1.1771 × 10^5^
	Std	6.4956 × 10^3^	2.0419 × 10^4^	9.2174 × 10^5^	1.5327 × 10^9^	4.8252 × 10^4^	2.0796 × 10^6^	3.0072 × 10^7^	9.8561 × 10^8^	3.5080 × 10^6^	4.8432 × 10^7^	1.1220 × 10^9^	1.3568 × 10^6^
	Avg	2.0695 × 10^4^	6.5280 × 10^4^	1.9703 × 10^6^	7.0808 × 10^8^	4.9100 × 10^4^	2.0921 × 10^6^	5.2702 × 10^7^	3.4995 × 10^9^	3.6268 × 10^6^	4.7574 × 10^7^	2.0164 × 10^9^	1.3305 × 10^6^
F20	Best	2.4762 × 10^3^	2.7141 × 10^3^	3.3086 × 10^3^	3.2021 × 10^3^	3.3570 × 10^3^	3.5142 × 10^3^	3.6365 × 10^3^	3.8484 × 10^3^	2.9029 × 10^3^	2.8701 × 10^3^	3.6320 × 10^3^	4.4451 × 10^3^
	Std	2.0461 × 10^2^	2.7918 × 10^2^	3.6721 × 10^2^	1.0901 × 10^2^	1.6425 × 10^2^	3.6151 × 10^2^	4.6725 × 10^2^	2.9750 × 10^2^	2.7759 × 10^2^	5.3669 × 10^2^	2.6302 × 10^2^	1.8211 × 10^2^
	Avg	2.7348 × 10^3^	3.1408 × 10^3^	3.7645 × 10^3^	3.2858 × 10^3^	3.5985 × 10^3^	4.1513 × 10^3^	4.1210 × 10^3^	4.1885 × 10^3^	3.1451 × 10^3^	3.5798 × 10^3^	3.9854 × 10^3^	4.6260 × 10^3^
F21	Best	2.3859 × 10^3^	2.5249 × 10^3^	2.7949 × 10^3^	2.8911 × 10^3^	2.6155 × 10^3^	3.0577 × 10^3^	3.0057 × 10^3^	3.1855 × 10^3^	2.4827 × 10^3^	2.7569 × 10^3^	2.9283 × 10^3^	2.8683 × 10^3^
	Std	3.0390 × 10^1^	1.7468 × 10^1^	1.1213 × 10^2^	1.0789 × 10^2^	9.5469 × 10^1^	4.4588 × 10^1^	8.4194 × 10^1^	6.1440 × 10^1^	4.8956 × 10^1^	6.2787 × 10^1^	4.3148 × 10^1^	6.3291 × 10^1^
	Avg	2.4115 × 10^3^	2.5455 × 10^3^	2.9331 × 10^3^	3.0010 × 10^3^	2.7061 × 10^3^	3.1196 × 10^3^	3.0925 × 10^3^	3.2483 × 10^3^	2.5363 × 10^3^	2.8097 × 10^3^	2.9752 × 10^3^	2.9333 × 10^3^
F22	Best	2.3236 × 10^3^	2.4033 × 10^3^	1.1758 × 10^4^	1.0584 × 10^4^	9.4005 × 10^3^	1.1020 × 10^4^	1.1704 × 10^4^	1.5701 × 10^4^	8.8965 × 10^3^	1.3226 × 10^4^	1.5292 × 10^4^	1.7404 × 10^4^
	Std	5.8338 × 10^0^	4.6388 × 10^3^	9.7444 × 10^2^	2.2062 × 10^3^	8.2419 × 10^2^	1.0725 × 10^3^	1.1480 × 10^3^	4.9985 × 10^2^	3.2495 × 10^3^	1.0313 × 10^3^	8.4940 × 10^2^	1.2294 × 10^2^
	Avg	2.3309 × 10^3^	5.7525 × 10^3^	1.2405 × 10^4^	1.2515 × 10^4^	1.0563 × 10^4^	1.1958 × 10^4^	1.3469 × 10^4^	1.6527 × 10^4^	1.1122 × 10^4^	1.4054 × 10^4^	1.6495 × 10^4^	1.7536 × 10^4^
F23	Best	2.8206 × 10^3^	2.9635 × 10^3^	3.8377 × 10^3^	3.5580 × 10^3^	3.4216 × 10^3^	3.8717 × 10^3^	3.5279 × 10^3^	4.3543 × 10^3^	2.9311 × 10^3^	3.4161 × 10^3^	3.7209 × 10^3^	3.3365 × 10^3^
	Std	1.2725 × 10^1^	2.0462 × 10^1^	1.4268 × 10^2^	1.6021 × 10^2^	2.2963 × 10^2^	3.2592 × 10^2^	2.5185 × 10^2^	1.8976 × 10^2^	1.4850 × 10^2^	1.4126 × 10^2^	1.2293 × 10^2^	7.5285 × 10^1^
	Avg	2.8302 × 10^3^	2.9978 × 10^3^	4.0310 × 10^3^	3.7580 × 10^3^	3.6823 × 10^3^	4.3089 × 10^3^	3.9198 × 10^3^	4.5585 × 10^3^	3.0736 × 10^3^	3.5061 × 10^3^	3.8621 × 10^3^	3.4169 × 10^3^
F24	Best	2.9705 × 10^3^	3.0667 × 10^3^	4.1026 × 10^3^	3.5928 × 10^3^	3.2994 × 10^3^	4.2914 × 10^3^	3.6099 × 10^3^	4.5603 × 10^3^	3.1294 × 10^3^	3.5972 × 10^3^	3.9954 × 10^3^	3.6356 × 10^3^
	Std	1.9687 × 10^1^	3.4636 × 10^1^	2.4826 × 10^2^	2.7099 × 10^2^	2.4848 × 10^2^	1.1787 × 10^2^	2.5736 × 10^2^	3.2709 × 10^2^	1.2895 × 10^2^	1.2867 × 10^2^	1.5910 × 10^2^	7.4568 × 10^0^
	Avg	2.9952 × 10^3^	3.1129 × 10^3^	4.3952 × 10^3^	3.8400 × 10^3^	3.6259 × 10^3^	4.4060 × 10^3^	3.9258 × 10^3^	4.9553 × 10^3^	3.2127 × 10^3^	3.7767 × 10^3^	4.2127 × 10^3^	3.6438 × 10^3^
F25	Best	3.0952 × 10^3^	3.1362 × 10^3^	3.5185 × 10^3^	4.2259 × 10^3^	3.3820 × 10^3^	3.2831 × 10^3^	4.2991 × 10^3^	1.4079 × 10^4^	3.4500 × 10^3^	4.4161 × 10^3^	9.4678 × 10^3^	6.7798 × 10^3^
	Std	3.9142 × 10^1^	2.1236 × 10^1^	2.5288 × 10^2^	3.5785 × 10^3^	2.4668 × 10^2^	1.1450 × 10^3^	9.8173 × 10^2^	1.1111 × 10^3^	2.1848 × 10^2^	8.7817 × 10^2^	1.3355 × 10^3^	1.3135 × 10^3^
	Avg	3.1404 × 10^3^	3.1645 × 10^3^	3.7505 × 10^3^	6.9976 × 10^3^	3.6307 × 10^3^	4.0471 × 10^3^	5.1461 × 10^3^	1.5818 × 10^4^	3.6954 × 10^3^	5.5958 × 10^3^	1.1594 × 10^4^	8.5612 × 10^3^
F26	Best	2.9419 × 10^3^	3.4019 × 10^3^	1.2210 × 10^4^	1.1723 × 10^4^	5.4648 × 10^3^	1.6964 × 10^4^	1.2257 × 10^4^	1.7032 × 10^4^	6.3724 × 10^3^	1.2285 × 10^4^	1.3121 × 10^4^	9.1502 × 10^3^
	Std	7.2702 × 10^0^	1.4705 × 10^3^	7.1417 × 10^2^	9.3059 × 10^2^	2.4386 × 10^3^	2.0910 × 10^3^	2.1888 × 10^3^	3.7426 × 10^2^	5.8813 × 10^2^	5.1048 × 10^2^	9.6559 × 10^2^	8.8620 × 10^2^
	Avg	2.9483 × 10^3^	4.2581 × 10^3^	1.2865 × 10^4^	1.2877 × 10^4^	8.3829 × 10^3^	1.9715 × 10^4^	1.5670 × 10^4^	1.7531 × 10^4^	6.7147 × 10^3^	1.3113 × 10^4^	1.4390 × 10^4^	1.0382 × 10^4^
F27	Best	3.3822 × 10^3^	3.4458 × 10^3^	4.1083 × 10^3^	3.8811 × 10^3^	3.6629 × 10^3^	5.0086 × 10^3^	4.3320 × 10^3^	6.7222 × 10^3^	3.6728 × 10^3^	3.9470 × 10^3^	4.3198 × 10^3^	3.2000 × 10^3^
	Std	3.2898 × 10^1^	9.8876 × 10^1^	3.9117 × 10^2^	3.0090 × 10^2^	3.6418 × 10^2^	5.4175 × 10^2^	6.2124 × 10^2^	2.8669 × 10^2^	1.2920 × 10^2^	3.9664 × 10^2^	5.0693 × 10^2^	9.1597 × 10^−5^
	Avg	3.4132 × 10^3^	3.5233 × 10^3^	4.5337 × 10^3^	4.1497 × 10^3^	3.9892 × 10^3^	5.9340 × 10^3^	4.8336 × 10^3^	7.1762 × 10^3^	3.7687 × 10^3^	4.2428 × 10^3^	5.1919 × 10^3^	3.2000 × 10^3^
F28	Best	3.3510 × 10^3^	3.3638 × 10^3^	4.3103 × 10^3^	5.1001 × 10^3^	3.8011 × 10^3^	4.0551 × 10^3^	5.4866 × 10^3^	1.2070 × 10^4^	4.3279 × 10^3^	5.5727 × 10^3^	8.7011 × 10^3^	3.3000 × 10^3^
	Std	2.3077 × 10^1^	6.1586 × 10^1^	5.1981 × 10^2^	3.9784 × 10^3^	3.8031 × 10^2^	3.7894 × 10^2^	3.7456 × 10^2^	9.2513 × 10^2^	3.0402 × 10^2^	5.6600 × 10^2^	8.3594 × 10^2^	1.1562 × 10^−3^
	Avg	3.3790 × 10^3^	3.4375 × 10^3^	4.9060 × 10^3^	7.3845 × 10^3^	4.2437 × 10^3^	4.4061 × 10^3^	5.9320 × 10^3^	1.3113 × 10^4^	4.7020 × 10^3^	6.2336 × 10^3^	9.7371 × 10^3^	3.3000 × 10^3^
F29	Best	3.5681 × 10^3^	4.3235 × 10^3^	6.1443 × 10^3^	5.7251 × 10^3^	4.9276 × 10^3^	7.6155 × 10^3^	7.3573 × 10^3^	1.7150 × 10^4^	4.5914 × 10^3^	5.9423 × 10^3^	9.0110 × 10^3^	8.9612 × 10^3^
	Std	2.1596 × 10^2^	4.1758 × 10^2^	9.1015 × 10^2^	2.0446 × 10^3^	5.7109 × 10^2^	1.1553 × 10^3^	1.7163 × 10^3^	3.3026 × 10^5^	2.0487 × 10^2^	1.2774 × 10^3^	2.4321 × 10^3^	1.0289 × 10^3^
	Avg	3.9089 × 10^3^	4.8473 × 10^3^	7.1085 × 10^3^	7.7002 × 10^3^	5.5354 × 10^3^	8.6532 × 10^3^	9.7369 × 10^3^	2.2088 × 10^5^	4.8739 × 10^3^	7.6033 × 10^3^	1.0944 × 10^4^	9.8484 × 10^3^
F30	Best	2.1291 × 10^6^	5.5870 × 10^6^	6.3975 × 10^7^	3.9051 × 10^7^	1.9471 × 10^7^	4.1713 × 10^7^	1.5459 × 10^8^	7.9346 × 10^9^	6.7575 × 10^7^	1.6582 × 10^8^	1.0683 × 10^9^	7.1611 × 10^7^
	Std	1.6597 × 10^6^	1.1988 × 10^7^	5.3742 × 10^7^	5.9747 × 10^8^	1.3068 × 10^7^	5.5680 × 10^7^	1.0661 × 10^8^	2.6467 × 10^9^	3.0970 × 10^8^	2.0705 × 10^8^	1.2712 × 10^9^	2.2264 × 10^8^
	Avg	3.9242 × 10^6^	1.8636 × 10^7^	1.3900 × 10^8^	3.3791 × 10^8^	3.8902 × 10^7^	9.6491 × 10^7^	2.7558 × 10^8^	1.0155 × 10^10^	2.5319 × 10^8^	3.4749 × 10^8^	1.8358 × 10^9^	3.2977 × 10^8^

**Table 3:** Wilcoxon rank-sum test results for ARGLO and the competing algorithms based on CEC2017 at dim = 50.

	Algorithms	GLO	HHO	BKA	PSO	GOOSE	WOA	COA	GWO	PO	DBO	DMOA
Functions												
F1		7.94 × 10^−3^	7.94 × 10^−3^	7.94 × 10^−3^	7.94 × 10^−3^	7.94 × 10^−3^	7.94 × 10^−3^	7.94 × 10^−3^	7.94 × 10^−3^	7.94 × 10^−3^	7.94 × 10^−3^	7.94 × 10^−3^
F3		1.59 × 10^−2^	9.52 × 10^−2^	7.94 × 10^−3^	1.51 × 10^−1^	7.94 × 10^−3^	7.94 × 10^−3^	7.94 × 10^−3^	1.59 × 10^−2^	6.90 × 10^−1^	7.94 × 10^−3^	7.94 × 10^−3^
F4		7.94 × 10^−3^	7.94 × 10^−3^	7.94 × 10^−3^	7.94 × 10^−3^	7.94 × 10^−3^	7.94 × 10^−3^	7.94 × 10^−3^	7.94 × 10^−3^	7.94 × 10^−3^	7.94 × 10^−3^	7.94 × 10^−3^
F5		1.51 × 10^−1^	7.94 × 10^−3^	1.59 × 10^−2^	1.51 × 10^−1^	7.94 × 10^−3^	7.94 × 10^−3^	7.94 × 10^−3^	1.51 × 10^−1^	7.94 × 10^−3^	7.94 × 10^−3^	7.94 × 10^−3^
F6		7.94 × 10^−3^	7.94 × 10^−3^	7.94 × 10^−3^	7.94 × 10^−3^	7.94 × 10^−3^	7.94 × 10^−3^	7.94 × 10^−3^	7.94 × 10^−3^	7.94 × 10^−3^	7.94 × 10^−3^	7.94 × 10^−3^
F7		7.94 × 10^−3^	7.94 × 10^−3^	7.94 × 10^−3^	7.94 × 10^−3^	7.94 × 10^−3^	7.94 × 10^−3^	7.94 × 10^−3^	6.90 × 10^−1^	7.94 × 10^−3^	7.94 × 10^−3^	7.94 × 10^−3^
F8		7.94 × 10^−3^	7.94 × 10^−3^	7.94 × 10^−3^	7.94 × 10^−3^	7.94 × 10^−3^	7.94 × 10^−3^	7.94 × 10^−3^	7.94 × 10^−3^	7.94 × 10^−3^	7.94 × 10^−3^	7.94 × 10^−3^
F9		7.94 × 10^−3^	7.94 × 10^−3^	7.94 × 10^−3^	7.94 × 10^−3^	7.94 × 10^−3^	7.94 × 10^−3^	7.94 × 10^−3^	7.94 × 10^−3^	7.94 × 10^−3^	7.94 × 10^−3^	7.94 × 10^−3^
F10		7.94 × 10^−3^	7.94 × 10^−3^	7.94 × 10^−3^	1.51 × 10^−1^	7.94 × 10^−3^	7.94 × 10^−3^	7.94 × 10^−3^	1.59 × 10^−2^	7.94 × 10^−3^	7.94 × 10^−3^	7.94 × 10^−3^
F11		7.94 × 10^−3^	7.94 × 10^−3^	7.94 × 10^−3^	7.94 × 10^−3^	7.94 × 10^−3^	7.94 × 10^−3^	7.94 × 10^−3^	7.94 × 10^−3^	7.94 × 10^−3^	7.94 × 10^−3^	7.94 × 10^−3^
F12		7.94 × 10^−3^	7.94 × 10^−3^	7.94 × 10^−3^	7.94 × 10^−3^	7.94 × 10^−3^	7.94 × 10^−3^	7.94 × 10^−3^	7.94 × 10^−3^	7.94 × 10^−3^	7.94 × 10^−3^	7.94 × 10^−3^
F13		7.94 × 10^−3^	7.94 × 10^−3^	7.94 × 10^−3^	7.94 × 10^−3^	7.94 × 10^−3^	7.94 × 10^−3^	7.94 × 10^−3^	7.94 × 10^−3^	7.94 × 10^−3^	7.94 × 10^−3^	7.94 × 10^−3^
F14		9.52 × 10^−2^	7.94 × 10^−3^	1.00×00	6.90 × 10^−1^	5.48 × 10^−1^	1.59 × 10^−2^	7.94 × 10^−3^	7.94 × 10^−3^	7.94 × 10^−3^	7.94 × 10^−3^	7.94 × 10^−3^
F15		7.94 × 10^−3^	7.94 × 10^−3^	7.94 × 10^−3^	9.52 × 10^−2^	1.00×00	7.94 × 10^−3^	7.94 × 10^−3^	7.94 × 10^−3^	7.94 × 10^−3^	7.94 × 10^−3^	7.94 × 10^−3^
F16		2.22 × 10^−1^	3.17 × 10^−2^	5.56 × 10^−2^	3.10 × 10^−1^	7.94 × 10^−3^	7.94 × 10^−3^	7.94 × 10^−3^	5.48 × 10^−1^	7.94 × 10^−3^	7.94 × 10^−3^	7.94 × 10^−3^
F17		1.59 × 10^−2^	7.94 × 10^−3^	7.94 × 10^−3^	7.94 × 10^−3^	7.94 × 10^−3^	7.94 × 10^−3^	7.94 × 10^−3^	7.94 × 10^−3^	7.94 × 10^−3^	7.94 × 10^−3^	7.94 × 10^−3^
F18		5.56 × 10^−2^	7.94 × 10^−3^	4.21 × 10^−1^	6.90 × 10^−1^	1.00×00	7.94 × 10^−3^	7.94 × 10^−3^	9.52 × 10^−2^	7.94 × 10^−3^	7.94 × 10^−3^	7.94 × 10^−3^
F19		7.94 × 10^−3^	7.94 × 10^−3^	7.94 × 10^−3^	8.41 × 10^−1^	7.94 × 10^−3^	7.94 × 10^−3^	7.94 × 10^−3^	7.94 × 10^−3^	7.94 × 10^−3^	7.94 × 10^−3^	7.94 × 10^−3^
F20		5.56 × 10^−2^	7.94 × 10^−3^	7.94 × 10^−3^	7.94 × 10^−3^	7.94 × 10^−3^	7.94 × 10^−3^	7.94 × 10^−3^	1.59 × 10^−2^	1.59 × 10^−2^	7.94 × 10^−3^	7.94 × 10^−3^
F21		7.94 × 10^−3^	7.94 × 10^−3^	7.94 × 10^−3^	7.94 × 10^−3^	7.94 × 10^−3^	7.94 × 10^−3^	7.94 × 10^−3^	7.94 × 10^−3^	7.94 × 10^−3^	7.94 × 10^−3^	7.94 × 10^−3^
F22		7.94 × 10^−3^	7.94 × 10^−3^	7.94 × 10^−3^	7.94 × 10^−3^	7.94 × 10^−3^	7.94 × 10^−3^	7.94 × 10^−3^	7.94 × 10^−3^	7.94 × 10^−3^	7.94 × 10^−3^	7.94 × 10^−3^
F23		7.94 × 10^−3^	7.94 × 10^−3^	7.94 × 10^−3^	7.94 × 10^−3^	7.94 × 10^−3^	7.94 × 10^−3^	7.94 × 10^−3^	7.94 × 10^−3^	7.94 × 10^−3^	7.94 × 10^−3^	7.94 × 10^−3^
F24		7.94 × 10^−3^	7.94 × 10^−3^	7.94 × 10^−3^	7.94 × 10^−3^	7.94 × 10^−3^	7.94 × 10^−3^	7.94 × 10^−3^	7.94 × 10^−3^	7.94 × 10^−3^	7.94 × 10^−3^	7.94 × 10^−3^
F25		4.21 × 10^−1^	7.94 × 10^−3^	7.94 × 10^−3^	7.94 × 10^−3^	7.94 × 10^−3^	7.94 × 10^−3^	7.94 × 10^−3^	7.94 × 10^−3^	7.94 × 10^−3^	7.94 × 10^−3^	7.94 × 10^−3^
F26		7.94 × 10^−3^	7.94 × 10^−3^	7.94 × 10^−3^	7.94 × 10^−3^	7.94 × 10^−3^	7.94 × 10^−3^	7.94 × 10^−3^	7.94 × 10^−3^	7.94 × 10^−3^	7.94 × 10^−3^	7.94 × 10^−3^
F27		1.59 × 10^−2^	7.94 × 10^−3^	7.94 × 10^−3^	7.94 × 10^−3^	7.94 × 10^−3^	7.94 × 10^−3^	7.94 × 10^−3^	7.94 × 10^−3^	7.94 × 10^−3^	7.94 × 10^−3^	7.94 × 10^−3^
F28		1.51 × 10^−1^	7.94 × 10^−3^	7.94 × 10^−3^	7.94 × 10^−3^	7.94 × 10^−3^	7.94 × 10^−3^	7.94 × 10^−3^	7.94 × 10^−3^	7.94 × 10^−3^	7.94 × 10^−3^	7.94 × 10^−3^
F29		7.94 × 10^−3^	7.94 × 10^−3^	7.94 × 10^−3^	7.94 × 10^−3^	7.94 × 10^−3^	7.94 × 10^−3^	7.94 × 10^−3^	7.94 × 10^−3^	7.94 × 10^−3^	7.94 × 10^−3^	7.94 × 10^−3^
F30		1.59 × 10^−2^	7.94 × 10^−3^	7.94 × 10^−3^	7.94 × 10^−3^	7.94 × 10^−3^	7.94 × 10^−3^	7.94 × 10^−3^	7.94 × 10^−3^	7.94 × 10^−3^	7.94 × 10^−3^	7.94 × 10^−3^

**Table 4:** Performance on the test set.

Metrics	SVR	GLO-SVR	ARGLO-SVR
MAE	2.326	1.565	**1.445**
MSE	9.024	4.275	**3.769**
RMSE	3.004	2.067	**1.942**
R^2^	0.73	0.87	**0.89**
RPD	1.933	2.81	**2.99**
MAPE	0.068	0.046	**0.042**

## Data Availability

The original contributions presented in this study are included in the article. Further inquires can be directed to the corresponding author.
